# Bridge-Induced Chromosome Translocation in Yeast Relies upon a Rad54/Rdh54-Dependent, Pol32-Independent Pathway

**DOI:** 10.1371/journal.pone.0060926

**Published:** 2013-04-17

**Authors:** Valentina Tosato, Sabrina Sidari, Carlo V. Bruschi

**Affiliations:** Yeast Molecular Genetics Laboratory, International Centre for Genetic Engineering and Biotechnology, Trieste, Italy; Texas A&M University, United States of America

## Abstract

While in mammalian cells the genetic determinism of chromosomal translocation remains unclear, the yeast *Saccharomyces cerevisiae* has become an ideal model system to generate *ad hoc* translocations and analyze their cellular and molecular outcome. A linear DNA cassette carrying a selectable marker flanked by perfect homologies to two chromosomes triggers a bridge-induced translocation (BIT) in budding yeast, with variable efficiency. A postulated two-step process to produce BIT translocants is based on the cooperation between the Homologous Recombination System (HRS) and Break-Induced Replication (BIR); however, a clear indication of the molecular factors underlying the genetic mechanism is still missing. In this work we provide evidence that BIT translocation is elicited by the Rad54 helicase and completed by a Pol32-independent replication pathway. Our results demonstrate also that Rdh54 is involved in the stability of the translocants, suggesting a mitotic role in chromosome pairing and segregation. Moreover, when *RAD54* is over-expressed, an ensemble of secondary rearrangements between repeated DNA tracts arise after the initial translocation event, leading to severe aneuploidy with loss of genetic material, which prompts the identification of fragile sites within the yeast genome.

## Introduction

Chromosomal translocations characterize many types of cancers [1], [2] and also can become useful markers in the diagnosis of liquid and solid tumors [3], [4]. However, the molecular factors that elicit this gross chromosomal rearrangement (GCR) are still object of investigation [5], [6].

*Saccharomyces cerevisiae* has been largely recognized as an ideal model organism to study genome instability [7]. Recently, our group developed an experimental system in yeast, named BIT, which allows the *ad hoc* generation of a chromosomal translocation between any two pre-selected genomic *loci* of diploid cells [8]. The method, which exploits the homologous recombination system of yeast, consists of the DNA transformation of diploid cells with a PCR-amplified selectable DNA fragment carrying at its ends sequences homologous to the chromosomal sites chosen for translocation. We discovered that this kind of bridge-induced translocation is non-reciprocal, that its frequency depends on the extension of the homology [8], that it occurs with similar efficiency between heterologous and homologous chromosomes [9] and that it is usually associated with the formation of viable aneuploids [10]. From these previous observations and from the transcriptome analysis of the translocants [11], we suppose that at least two pathways are playing a role in the initial steps of the BIT process: a Rad52 epistasis group-dependent homologous recombination (HR) and a break-induced replication. Therefore, we postulated a two-step independent integration event, according to the model of chromosomal fragment formation [12]. By this assumption, the Rad52-HRS should be responsible for the double strand break (DSB) repair resulting first in the integration of one of the free ends of the linear DNA molecule that is distal with respect to the centromere. In the second step, BIR may produce a partial trisomy and regulate translocation efficiency depending on the distance of the other DNA free end from the telomere. In this view, the BIT system resembles the gene-targeting (GT) tool, although the targets belong to two different chromosomes. In GT, the Rad52-epistasis group of genes are fundamental, but with different roles in budding yeast and in animal cells. For instance, in *S. cerevisiae, RAD52* is essential for GT while *RAD51* is playing a minor role with a 8-fold reduction of homologous integration when it is mutated [13]. On the contrary, in animal cells, GT depends on *RAD51* whereas *RAD52* affects less the gene-knock out (KO) process and it is not required for the repair of induced DSBs [14]. Moreover, *RAD51* deletion results in cell unviability in vertebrate and early embryonic death in mice [15], [16] while the same gene is not even haploinsufficient in yeast. Among the HRS genes (see [17] for a detailed review), *RAD54* has a critical role as the processive motor protein that translocates on dsDNA during DSB repair, recombination and GT. Its chromatin remodeling, bidirectional branch migration and post-synaptic functions seem to be conserved in human and yeast orthologues [18]. Furthermore, yeast *RAD54* over-expression in higher eukaryotes enhances GT [19] and, in contrast to *RAD51*, its absence is absolutely compatible with normal mouse development [17]. Point mutations in *RAD54A* or in its homologue *RAD54B* in human cells are associated with many types of tumors [20] and the unbalanced expression of *RAD54A* is associated with cancer progression [21], [22] in malignant cell lines (such as LNCaP, DU-145, PC-3) characterized by non-reciprocal chromosomal translocations [23], [24]. Important for long-homology GT [25], *RAD54* is dispensable for other DSBs repair pathways such as BIR [26], which can be *RAD51*-independent as well, but it requires the non-essential DNA polymerase delta subunit Pol32 [27].

In this work we investigated the putative molecular players that may either promote or impair the two-steps of the BIT event, focusing particularly on Rad54 (Rad54A) and Rdh54 (Rad54B) for the HRS and on Pol32 for BIR. We also compared BIT efficiency with the type of rearrangements formed during translocation among different null mutants of genes involved in different aspects of genome stability and, in particular, in translocation-associated recombination, such as *SGS1* [28], *TOP1* [29], [30], *XRS2* [31], *ELG1* [32], *MSH2* [33]. We found that *RAD54* was crucial for the outcome of BIT and that *RDH54* has a key role not only in BIT between heterologous chromosomes, where it is involved in the control of genome stability, but also in translocation between homologues, where it seems to have a role in the Holliday junction resolution process. Moreover, in complete absence of Pol32 BIT is still occurring, although in a reduced number of surviving cells, generating translocants with partial trisomy, which suggests that the process of DNA replication to complete the bridge is mostly Pol32-independent. We finally demonstrated that, after the formation of DSBs due to a BIT event, the over-expression of *RAD54* triggers secondary rearrangements between long-terminal repeats (LTRs) proximal to the breaks, producing an ensemble of further multiple translocations. This discovery could yield insights into the key role of the HR player *RAD54* in break-distal rearrangements between repeats, which are landmark traits of tumor cells.

## Results

### BIT in Recombination Mutants

Both copies of *RAD54* and of *RDH54 (TID1),* components of the Rad52-epistasis group, and of *POL32,* the pivotal gene of the BIR pathway, were deleted following the pop-in/pop-out technology (see Materials & Methods). Also the double deletants of other five genes (*ELG1, MSH2, SGS1, TOP1, XRS2*), which are considered fundamental in translocation-associated recombination, were generated as well. At first, to understand the influence of the selected genes on DNA transformation and on GT *per se*, the mutants were tested for their ability to be transformed by a circular plasmid (YCp50) (Table S1) and by a linear DNA cassette for the targeted KO of the gene *DUR3* (Fig. S1), respectively. *DUR3,* which codes for a polyamine transporter, was also one of the two genes targeted in the BIT experiments. All the data concerning the frequencies of transformation and integration of the mutants analyzed were calculated relative to the susceptibility of the mutants to be transformed, that is, to uptake DNA and transport it into the nucleus. The transformability of these strains was assessed by their transformation with the circular plasmid YCp50 (see Materials and Methods). The transformants obtained with linear DNA were screened on both integration sites using a three-primers colony-PCR strategy [8] in order to avoid that a lack of PCR product could be considered as absence of integration (ectopic DNA integration). For ectopic transformants, one band only instead of two was recovered (not shown, [8]) using the primers reported in Fig. S1. The positive transformants were furthermore screened with the external primers on genomic DNA. The frequency **νp** (p = plasmid) reported in Table S1 represents the transformability of the mutants and it is calculated as number of transformants on –URA medium per number of cells treated with the YCp50 plasmid. For each mutant and for the wild type, **νt** (Table S1) represents the frequency of transformants obtained on G418, using the linear DNA cassette for the GT of *DUR3* (Fig. S1) while **νko** is the actual frequency of deletion of the gene *DUR3*. These values were divided for each strain by its **νp** (assessed with YCp50). Since the data are reported in Fig. S1 as related to strain transformability, mutants like *xrs2Δ/xrs2Δ* show a strong hyper-rec phenotype with linear DNA cassettes. This is due to the fact that *this* mutant shows a very poor transformability with plasmid DNA respect to the wild type (see νp in Table S1). In particular, mutants of *XRS2* and of *SGS1* are about thirty-fold less transformable with DNA than the wild type strain. Moreover, transformations with a KO DNA cassette of *elg1Δ/elg1Δ*, *sgs1Δ/sgs1Δ* and *xrs2Δ/xrs2Δ* generate high numbers of ectopic transformants (Fig. S1). This is in agreement with the reported hyper-recombination phenotype of these mutants [34], [35], [36] and with the possibility that spontaneous DSBs may occur in their genome, as already proposed for *elg1* [36]. Therefore, unrepaired DNA lesions following stalled replication forks [36], and their accumulation in G1 [35] may favor random integrations of linear DNA molecules regardless to their homology (Fig. S1). Also the *RAD54* and *RDH54* (but not *POL32*) deletants reveal a predictable, but moderate decrease in targeted integration respect to the total of transformation events (Fig. S1). However, from data of Table S1, it seems that the *RAD54* deletant is slightly hyper-rec respect to the wt and previous experiments reported that GT in *rad54* null mutants is reduced, but it is not absent [25]. Moreover, it was demonstrated that in *rad54* diploids the GT works better than in *rad54* haploids [25] and that the transformability of *rad54* diploids with a circular plasmid is at least 2-fold less than the wild type [25] consistently with our data (TableS1). The difference in GT (νko) that we observed between the wt and the *rad54Δ/rad54Δ* can be due to the shorter homology utilized for targeting (65 nt instead than 300 nt [25]).

**Figure 1:**
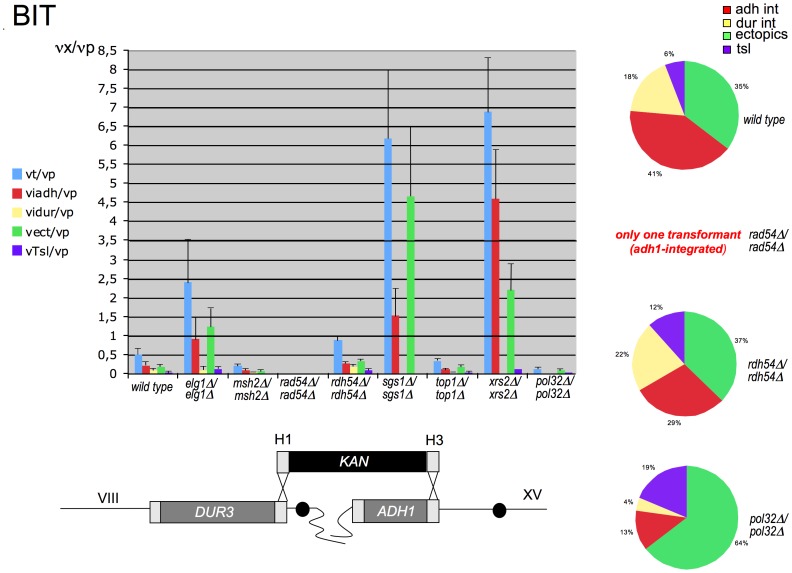
Frequency of recombination events with a BIT cassette in eight different mutants. The frequency (**ν**) of each genetic event **x** (transformation: **νt**; ectopic integration: **νi**ect; one-end DNA integration: **νiadh** and **νidur3**; translocation: **νTsl**) in the wild type and in eight mutants (see figure legend on the left) divided by the transformability of the strain (**νp**, Table S1). The frequency values **νx**/**νp** and the relative standard errors (here represented as plus bar only for graphical reasons) are summarized in Table S2b. **νx** values are calculated as total number of transformants obtained on G418 per event divided by the total amount of treated cells (raw data are in Table S2a). For details of the computation see Materials and Methods. To better interpret the data of Table S2a, the ratio distribution of the various integration events for *RAD54*, *RDH54*, and *POL32* mutants and for the wild type, from the total number of transformants, is plotted graphically as percentage pie charts. For the *rad54Δ/rad54Δ* mutant no pie has been drawn since only one transformant has been recovered, integrated only at the *adh1 locus.* A schematic representation of the BIT cassette with the two homologies targeting chromosomes VIII and XV is also shown at the bottom of the figure. As negative control, in *rad52Δ/rad52Δ* background only three ectopic clones were obtained, one coming from GT and two from BIT. The transformability of *rad52Δ/rad52Δ* with the centromeric plasmid YCp50 was assessed to be 1/6 of the wild type and therefore does not justify the low number of transformants obtained with a linear DNA cassette (Table S1 and S2). Thus, the deletion of *RAD52* completely abolishes the possibility to get any kind of targeted DNA integration confirming that BIT is HRS-dependent.

When we repeated the experiments using a BIT cassette, instead than a KO cassette, the results were remarkably different (Fig. 1, Table S2). This cassette has one homology (H1, coordinates 74204–74268) identical to the DNA cassette used to perform the KO of *DUR3* and a second homology (H3, coordinates 160613–160667) for the *adh1 locus* of chromosome XV (the BIT and KO cassettes are represented at the bottom of Fig. 1 and Fig. S1, respectively). Therefore, the BIT cassette carries at its ends two sequences homologous to two different chromosomes. Both genes (*ADH1, DUR3*) that were chosen as recombination targets *loci* are metabolic genes without any known effect on genetic recombination.

Several transformations were performed with each mutant (Table S2a) and a total of 406 transformants from all the strains were analyzed. Translocants (named AD) were formed via BIT in the wild type San1 with a frequency of 0.29×10^−8^ [3/(3×3.46×10^8^)] and stably maintained in the cell population, in full agreement with previous results [8]. In the wt and in the mutants, we focused on the formation of the AD bridge regardless the secondary rearrangements of the other fragments that may vary from one translocant to another within the same strain [9]. We analyzed the capacity of the cassette to: i) transform the wt or the mutant strain (**νt**), ii) to integrate at the adh1 site-only (**ν** ***iadh***), iii) to integrate at the dur3 site-only **(ν** ***idur3***), iv) to integrate ectopically **(ν** ***iect***) and, v) to generate verified translocants **(νTs** ***l***) (for **ν** values computation see Materials & Methods). Among all the deletants analyzed, *rad54Δ/rad54Δ* and *pol32Δ/pol32Δ* showed very low transformation frequency (Fig. 1, P<0.05) when compared to the wild type. For *rad54Δ/rad54Δ* the transformation frequency with the BIT cassette was below the detection level of any other transformant. This data are very different from what observed in GT experiments where, despite an expected reduction in targeting and the poor transformability of the mutant (Table S1), transformations were still possible with good frequencies (Fig. S1, Table S1). The deletion of *POL32*, despite the very low transformation frequency with the BIT cassette, still allows the formation of translocants. On the contrary, Pol32 does not have any effect on the GT (Fig. S1). Again, as for the KO experiment (Fig. S1), also with BIT there is a strong bias for ectopic integration in *elg1Δ/elg1Δ* (P<0.05), *sgs1Δ/sgs1Δ* (P<0.05) and *xrs2Δ/xrs2Δ* clones (0.05<P<0.1) (Fig. 1). Moreover, *sgs1Δ/sgs1Δ* and *xrs2Δ/xrs2Δ* did not show any integration at the *dur3 locus* of the BIT cassette (P<0.05). With respect to the distribution of integration events, *rdh54Δ/rdh54Δ* is the most similar to the wild type. To verify the hypothesis that integration events are Rad52-dependendent, a *rad52Δ/ra52Δ* strain was used (see Legend of Fig. 1 and Tables S1 and S2 for the raw data, P<0.05). To draw some significant general conclusions, it would not be possible to focus only on the frequency of verified translocants (**ν** ***Tsl*** value), since translocations via BIT are usually rare events (8,9). However, among all the mutants analyzed, *rad54Δ/rad54Δ* is the only one that strongly affects with good reproducibility (Table S2, P<0.05) not only translocations, but all kinds of integration events (one-end, two-ends integrations and ectopic - for a scheme of the different outcomes see Fig. S1) during BIT. For that reason, the role of *RAD54* in the process of BIT events was further investigated.

### *RAD54* Complementation and Over-expression

To confirm the data obtained with the *rad54Δ/rad54Δ* mutant, the *RAD54* gene was amplified (with its own promoter and terminator), cloned and sequenced in a pMOS vector and then transferred to the single-copy shuttle plasmid pRS416 (see Materials & Methods) for complementation of the function. Moreover, in order to verify whether an excess of Rad54 was affecting the BIT outcome, we decided to over-express the gene cloning it in the multi-copy plasmid PRS426 (see Materials & Methods). Both constructs were used to transform the homozygous deletant *rad54Δ/rad54Δ* and the effective expression and over-expression of the *RAD54* gene, (although not the protein expression level) was assessed by RT-PCR and quantified by densitometric readings (Fig. S2A). The expression of the gene, although not completely rescued to the wild type level, probably as a result of the influence of the surrounding DNA context, was mostly restored and translocants were again recovered (Fig. S2B). Indeed, the particular genomic location of a gene, whether on the chromosomes or on a plasmid, can affect the overall level of its expression, due to differences in the chromatin context [37] or to the instability of the plasmid carrier in non selective medium, respectively [38]. BIT was also successfully performed in the strain over-expressing *RAD54* (OeRAD54). The experiments were then performed in a heterozygous *RAD54/rad54Δ* strain demonstrating that the gene is fully dominant (Fig. S2B).

We moreover tested an excess of the AD cassette in *rad54Δ/rad54Δ* mutant to “force” the transformation, but we found that even increasing more than 3-fold (up to 30 ug) the amount of the linear DNA, the transformation efficiency in *rad54Δ/rad54Δ* is still 4–5 times less than with the wild type (data not shown). As general conclusion, we demonstrated that the translocation and even the one-end integration in *DUR3 locus* (Fig. 1), are extremely not favored in *rad54Δ/rad54Δ* mutants.

### Characterization of the Translocants

**Figure 2:**
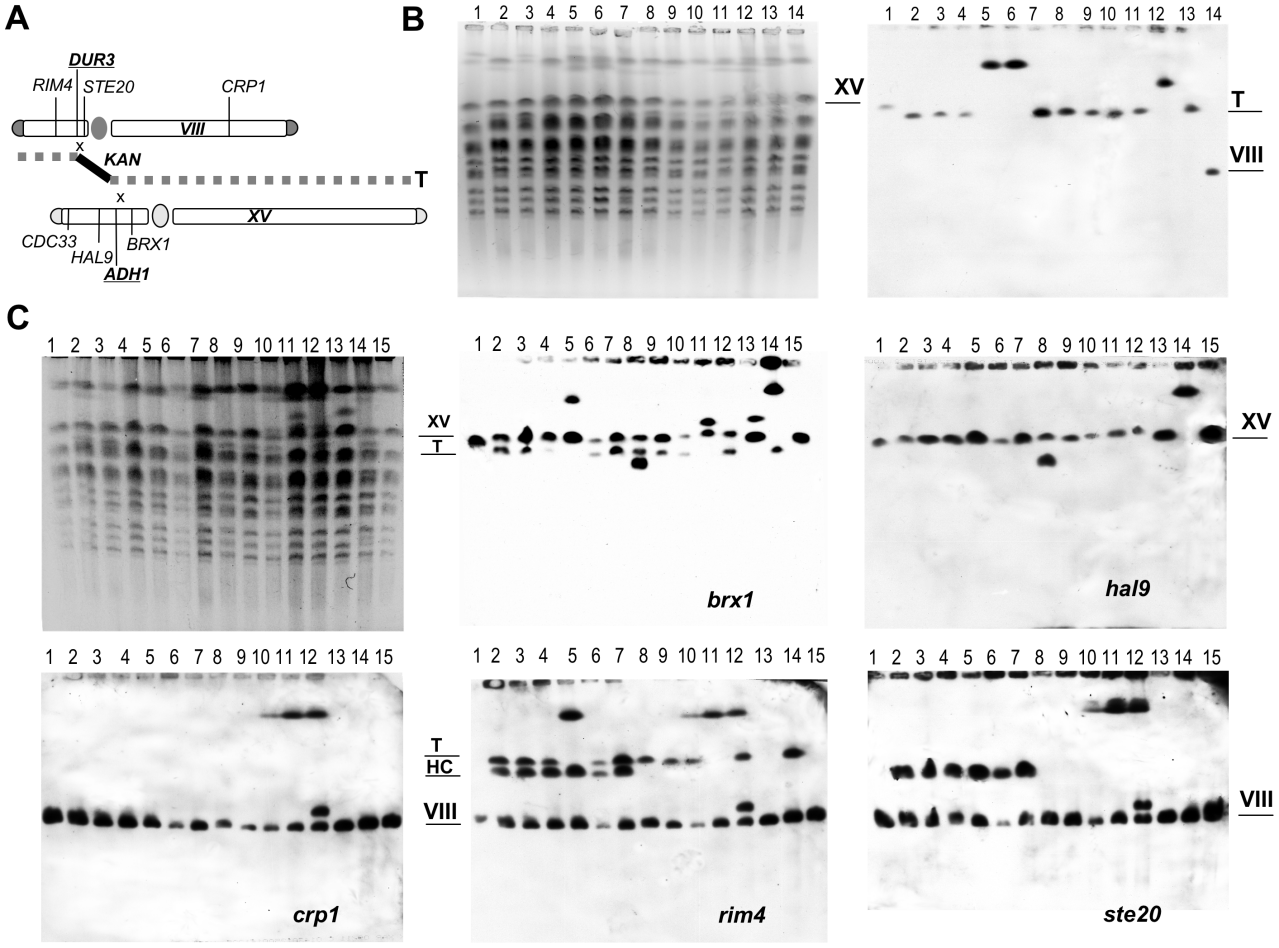
CHEF and Southern hybridization analysis of translocants unbalanced for Rdh54 and Rad54 amount. A) A scheme showing the bridge between chromosome VIII and XV and the new hybrid chromosome (Translocant = T). The chromosomal positions of the probes and of the translocation breakpoints (bold and underlined) are reported. B) CHEF (left) and Southern hybridization (right), with a probe against kanamycin, of the translocants in *RDH54* and *RAD54* deletants. Lanes from left to right: 1, San1ΔMSH2::Kan (it indicates chromosome XV); in *rdh54Δ/rdh54Δ* background: 2, cl4; 3, cl7; 4, cl16; 5, cl23n; 6, cl23*; 7, cl30; 8, cl47; in CRAD54 background: 9, cl1; 10, cl4; in OeRAD54 background: 11, clw; 12, cl37; 13, cl38; 14, San1ΔDUR3::Kan (chromosome VIII). n means phenotypically normal and * indicates sectored colony (see text for details). C) CHEF and Southern bots of the following samples (from left to right): 1, San1; in *rdh54Δ/rdh54Δ* background: 2, cl4; 3, cl7; 4, cl16; 5, cl23; 6, cl30 and 7, cl47. In CRAD54 background: 8, cl1 and 9, cl4; in OeRAD54 background: 10, clw; 11, cl37 and 12, cl38; 13, *top1Δ/top1Δ*cl18; 14, *xrs2Δ/xrs2Δ*cl6; 15, San1, hybridized with probes for *brx1, hal9* (upper line) and *cdc33* (not shown because identical to *hal9*) for chromosome XV and with *crp1*, *rim4* and *ste20* for chromosome VIII (bottom line). Two clones, obtained in *TOP1* and *XRS2* deletants, were also included in the CHEF (lanes 13 and 14); *brx* and *hal9* hybridization disclosed the complete loss of the native chromosome XV in *xrs2Δ/xrs2Δ*cl6 (lane 14).

The genomic DNA of translocants obtained in the wild type background of San1 and in the *RAD54* and *RDH54* deletants was run on a CHEF and hybridized with a probe against kanamycin (Fig. 2). The majority of the translocants showed the new translocated chromosome of the expected size of 1006504 nt, deriving from the fusion between chromosome XV and VIII catalyzed by the kanamycin cassette. The size of the translocated chromosome in clone 23 (*rdh54Δ/rdh54Δ*cl23, Fig. 2B, lanes 5, 6) and in OeRAD*54*cl37 (cl37, lane12) is bigger than expected suggesting further rearrangements at other genomic locations. This kind of event was previously noticed in translocations involving other pairs of non-homologous chromosomes and it is probably due to a BIR-dependent template switching-like mechanism [10]. *rdh54Δ/rdh54Δ*cl23 presented phenotypically smooth, normal colonies and star-like, lethal-sectoring colonies (Fig. 2B, lanes 5, 6); for this reason the two sub-populations were separated and independently analyzed, although they did not reveal any genomic difference between them. In Fig. 2C a membrane containing the same samples as in Fig. 2B plus two clones obtained in *TOP1* and *XRS2* mutant background, was stripped and re-hybridized with five probes along both chromosomes involved in the translocation event, to analyze their structure. The hybridization with the probe for *brx1* (Chr. XV, Fig. 2C) confirmed for all the samples the pattern indicated by the probe against kanamycin and revealed the complete loss of the native chromosome XV in *xrs2Δ/xrs2Δ*cl6 (cl6, lane 14). Moreover, one of the two clones in which *RAD54* is complemented (C*RAD54*cl1) presented a rearrangement of part of chromosome XV (Fig. 2C, cl1, lane 8). Similar rearrangements of a small portion of the left arm of chromosome XV were verified also in four of these translocants (*rdh54Δ/rdh54Δ*cl16, C*RAD54*cl1, Oe*RAD54*cl37, Oe*RAD54*cl38) by hybridization with a *cdc33* probe (data not shown).

#### *OERAD54* translocants

When *RAD54* is over-expressed (Fig. 2C lanes 10: clw, 11: cl37, 12: cl38), the entire chromosome VIII (or at least the majority of it) is probably duplicated and rearranged with a large chromosome (probes *crp1*, *rim4* and *ste20)*. Moreover, among them, cl38 (lane 12) exhibited a further rearrangement of chromosome VIII while cl37 (lane11) did not have the expected translocated chromosome, but a rearranged one. Amplification and sequencing of the junctions revealed an extensive duplication error on the *dur3* side (Fig. S3) and two point mutations (1 C deletion and 1 pyrimidine swap) within the *adh1* junction (data not shown). In addition, clone 37 was characterized by pseudo-hyphal growth, heavy flocculation and strong mortality (Fig. S3 A, B, C and D). Nevertheless, in cl37 as in the other translocants, there is no consistent correlation between phenotypic defects and DNA base alterations around the breakpoints (Fig. S3).

#### rdh54Δ/rdh54Δ translocants

From hybridizations along chromosome VIII it was also possible to detect a rearrangement of the left arm of this chromosome in all the *rdh54Δ/rdh54Δ* translocants producing a hybrid chromosome (HC, Fig. 2C, panels *rim4* and *ste20*). This HC was found to be present in the mutant prior BIT transformation and it was generated probably during the second pop-out, after the deletion of both alleles of *RDH54*. Unfortunately, this was the only *rdh54Δ*/*rdh54Δ* clone obtained after the pop out; the HC was probably due to the hyper-recombinagenicity of the mutant or to the mutagenicity of each transformation. The propensity of the mutant to recombine was confirmed also by the difficult construction of stable strains over-expressing *RDH54* starting from *rdh54Δ*/*rdh54Δ* (D. Nikitin, personal communication). Therefore, to understand whether this spontaneous rearrangement could affect BIT efficiency, we decided to investigate further this mutant. Serial hybridizations were performed walking along chromosome VIII (*loci tda3, kic1, ans1, dcd1, and cdc23*) resulting in the identification of the breakpoint within the right arm of the chromosome and in particular between the *dcd1* and *crp1 loci* (Fig. S4A). The quantitative densitometric readings of the hybridization bands (represented as histograms) confirm the loss of a fragment of the right arm of chromosome VIII in *rdh54Δ*/*rdh54Δ* (Fig. S4A). Given the large size of the HC (estimated approx. 880,930 kb from a linear regression curve, not shown), we decided to test the hypothesis that one of the largest chromosomes of yeast could be the other partner in the rearrangement. In particular, chromosome IV gave two bands after hybridization with *swr1* and *ydr262w* but not with *rad9* suggesting that the breakpoint was around 1 Mb from the left telomere (for details see Legend of Fig. S4A). The resulting HC was stably maintained in the *rdh54Δ/rdh54Δ* and in all the derivative XV–VIII translocants. However, its presence did not affect either the outcome of the translocation or the stability of the translocants, that was assessed to be very different depending on the particular translocant analyzed as presented further on in this work.

### The Over-expression of *RAD54* Promotes Secondary Translocations

**Figure 3:**
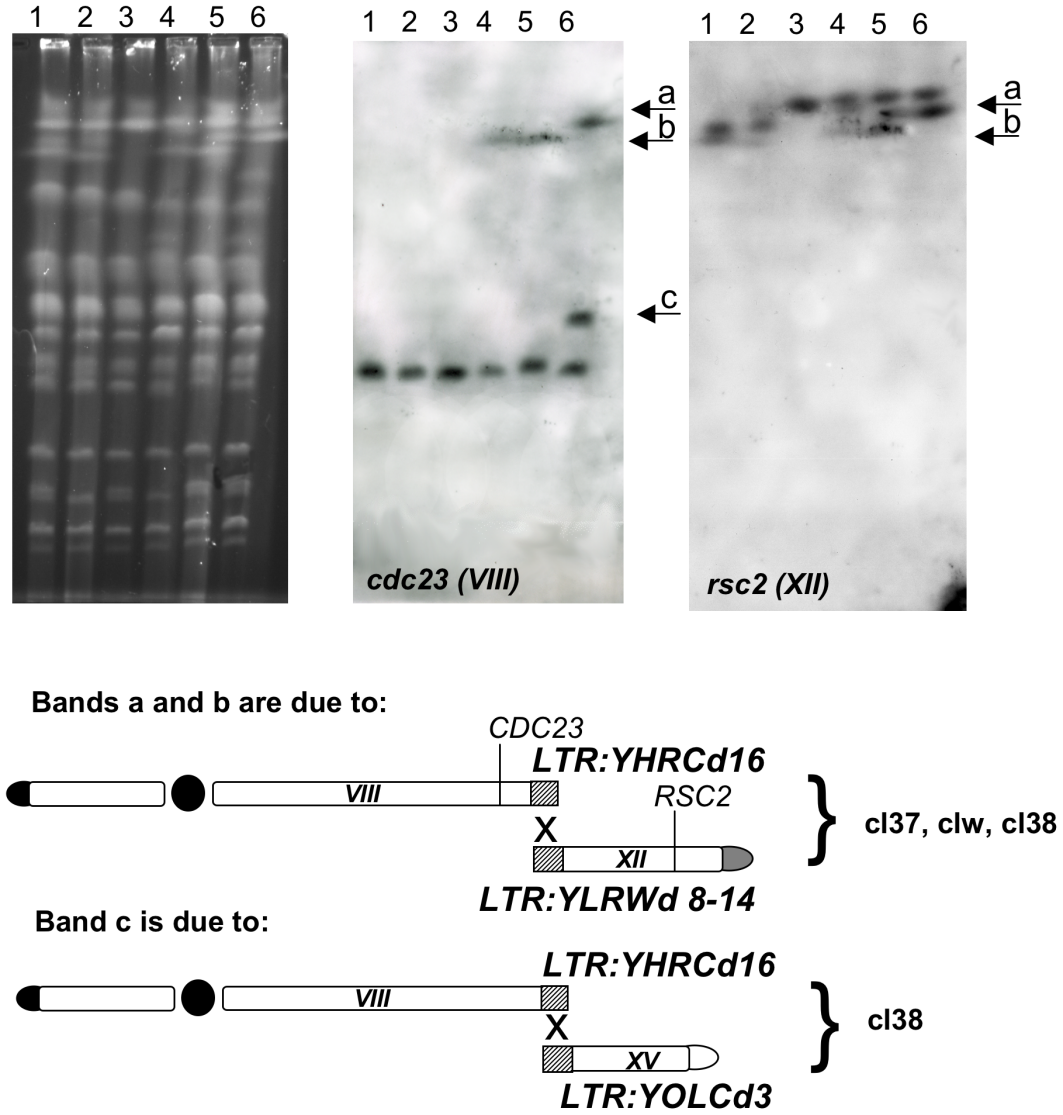
CHEF Southern hybridization using probes against the right arms of chromosomes VIII and XII. 1: wild type San1, 2: *rad54Δ/rad54Δ*, 3: OeRAD54, 4: OeRAD54clw, 5: OeRAD54cl37, 6: OeRAD54cl38. The hybridization with *cdc23* (VIII) points out the unexpected bands (a, b, c) in the translocated strains (lanes 4, 5, and 6) that are also probed with *rsc2* (XII). The same pattern obtained with *cdc23* was verified with probes against *rim4*, *rps20*, *ydr213* and *arn2* (not shown). A scheme of the recombination between LTRs explaining the bands a, b and c of the hybridizations is provided at the bottom.

As reported before, the over-expression of *RAD54* halves, but never abolishes, the likelihood of BIT. However, all the AD translocants obtained when *RAD54* is over-expressed share the peculiarity to bear an ensemble of rearrangements and unknown aneuploidies. Despite the fact that, among the 107 transformants, we could find and characterize only three *OeRAD54* translocants (Fig. S2B), the GCR-carrying genotype of these translocants was never detected either in the 23 translocants analyzed in this work or in other BIT translocants recovered and described in the past years (9, 10, 11). In the three *OeRAD54* translocants (clw, cl37, cl38), several bands appear after Southern blot hybridization with probes along chromosomes VIII and XII (*crp, rim, ste* in Fig. 2C; *cdc23* and *rsc2* in Fig. 3). The translocated chromosome is stable after replica plating (data not shown) although in cl 37 is rearranged (Fig. 2B, lane 12). In all these translocants a copy of almost the whole chromosome VIII is further rearranged with a larger chromosome and furthermore, only in cl38, another copy of the same chromosome VIII is rearranged with the fragment of chromosome XV distal to the DSB induced by BIT. A full summary of the rearrangements deduced from twelve different hybridizations is presented in Fig. 3. Unexpectedly, the rearrangements occurring on chromosome VIII after a break within the right arm localize very far from *DUR3.* We were able to precisely map the recombination points and we noticed that in all cases they consist of almost identical ΔLTRs (Fig. 3). In particular, two LTRs distal to the rDNA cluster of chromosome XII (YLRWΔ14 for cl37 and clw and probably YLRWΔ8 for cl.38, Fig. 3, lanes 4,5 and 6) one LTR on the pre-telomeric sequence of chromosome VIII (YHRCΔ16) and one LTR (YOLCΔ3) next to the *adh1* locus of chromosome XV were most likely responsible for these secondary translocations (Fig. 3). The exact identification of the LTR next to the rDNA cluster responsible for the rearrangement with chromosome VIII in cl38 is very difficult, due to the overlapping of the bands of the translocant with the band of the wild type chromosome XII. On the other hand, the 6.5 Kb bridge between XV and VIII in cl38 was easily amplified and randomly sequenced. It demonstrated that the fragment of chromosome XV was degraded from the DSB within *adh1* up to the LTR YOLΔ3 and then it recombined with the LTRYHRCΔ16 of chromosome VIII (Fig. 3). The breakpoints within the chromosomes VIII, XV and XII (shown in Fig. 3) and the integrity of chromosome IV were further confirmed with several hybridizations using primers listed in Table S3. These breakpoints, with the exception of the one in the left arm of chromosome XV, are not direct consequences of the BIT event. A possible explanation of their generation is presented in the discussion.

To better understand if the presence of LTRs was necessary for these further rearrangements and if the expression of *RAD54* affects BIT frequency regardless the targeted *loci*, we decided to repeat the experiment using the cassette named SUSU previously utilized to bridge chromosome IX and XVI [10]. In this case, the site *suc2* on chromosome IX where the cassette should integrate is very far away from any LTR (the nearest is located at 160 kb from *suc2*). Repeating the experiment in the case of over-expression of *RAD54* we obtained only 1 translocant (cl48) out of 91 transformants, meaning that the frequency of the event is nearly 1/10 than in the wild type [10] and confirming the importance of a stoichiometric amount of Rad54 for BIT efficiency. However, analyzing cl48 with probes against chromosome IX, XVI, VIII and kanamycin, only the expected translocated chromosome IX/XVI was present without any other apparent aneuploidy (Fig. S5 lane 3) or secondary rearrangement. This result suggests that, in this case, the fragment deriving from chromosome IX cannot recombine, due to the absence of distal LTRs. It might also imply that when the HRS is stimulated through the *RAD54* over-expression, aneuploidies of few, small chromosomes (such as VIII) are better tolerated than others by the yeast cell and this hypothesis could justify the verified absence of surviving cells carrying multi-copies of the rearranged, large chromosome XVI.

### Stability of the Translocated Chromosomes in *rdh54Δ/rdh54Δ Strains*

**Figure 4:**
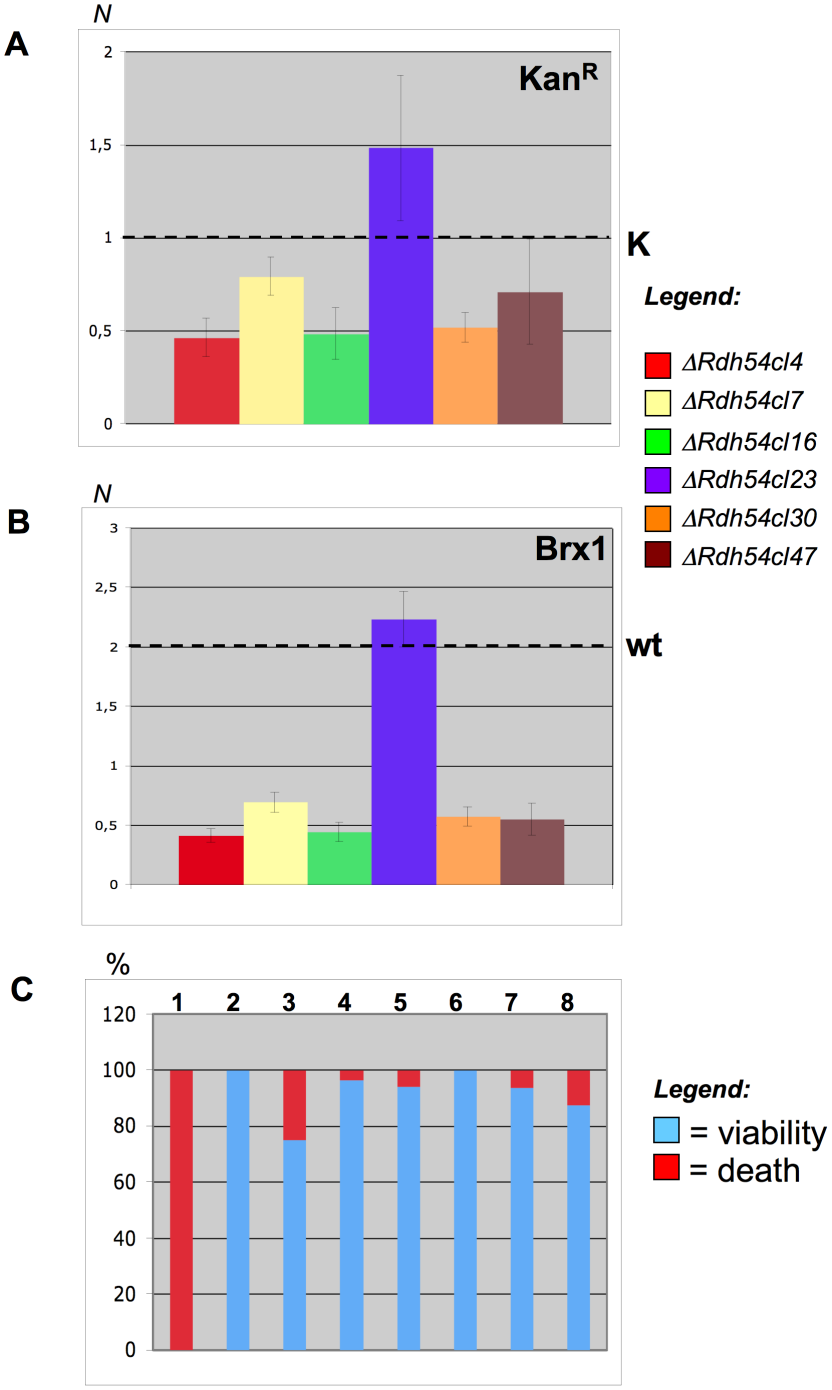
Chromosome stability in *rdh54Δ/rdh54Δ* translocants. A) Kanamycin gene copy number ( = *N*) obtained by quantitative PCR in the six *rdh54Δ/rdh54Δ* translocants compared with the control (K). The control K is a strain where one copy of kanamycin stably replaces the *DUR3* gene. B) Gene copy number ( = *N*) of *BRX1* in *rdh54Δ/rdh54Δ* translocants compared with the wild type (that has two copies of this gene). *BRX1* is located within chromosome XV (see Figure 2A) and on the translocated chromosome XV–VIII. Each histogram is the result of nine independent readings. C) Loss (%) of the chromosome carrying kanamycin, indicated by lack of growth on G418 medium (in red), versus viability (in blue) derived by replica-plating. The strains were grown overnight and plated without selection and then replicated on G418. The results are expressed in percentage. 1: wild type strain, 2: K, 3: *rdh54Δ/rdh54Δ*cl4, 4: *rdh54Δ/rdh54Δ*cl7, 5: *rdh54Δ/rdh54Δ*cl16, 6: *rdh54Δ/rdh54Δ*cl23, 7: *rdh54Δ/rdh54Δ*cl30, 8 *rdh54Δ/rdh54Δ*cl47.

Typically, the hybrid chromosomes that arise from a BIT translocation are quite stable also after several generations [8]. In rare cases they can undergo some rearrangement, such as shortening, because of sporadic Ty intra-chromosomal recombination [8] or fusions with other chromosomes [10]. Among the genes analyzed in this work, only *RDH54* seems to play a role in the stability of the translocants. Indeed, five out of six of the translocants generated in *rdh54Δ/rdh54Δ* background are lost with variable percentage. This loss was assessed using three independent sets of experiments based on the detection of gene copy number and replica plating (Fig. 4A, B and C). The loss of kanamycin (Fig. 4A and C) indicates instability of the translocated chromosome that is carrying the centromere of chromosome XV. Fig. 4B confirms this instability and suggests a further segregation defect of chromosome XV. It is peculiar that the only clone (cl23) that stably maintains the translocated chromosome after several generations is the one where the translocated chromosome is rearranged and bigger than expected (Fig. 2B lane5 and 6 and Fig. 2C, lane 5 with *brx1* and *rim4*). It was indeed verified that in translocant cl23 a fusion with another chromosome occurred and that the last 500 kb of the right arm of chromosome XV are missing (data not shown). However, this rearranged translocant, spontaneously selected, seems much more stable in absence of Rdh54 than the original one that we induced via BIT. The unusual behavior of clone 23 could also be due to an endo-reduplication of the rearranged translocated chromosome (as also supported by the number of copies of kanamycin resistance gene in Fig. 4A). Replica plating experiments and quantitative copy number tests on the remaining translocants described in this work indicated that the other genes analyzed were not correlated with the stability of the translocant (data not shown).

### Rdh54 Affects Homologous, but not Heterologous Translocation Events

**Figure 5:**
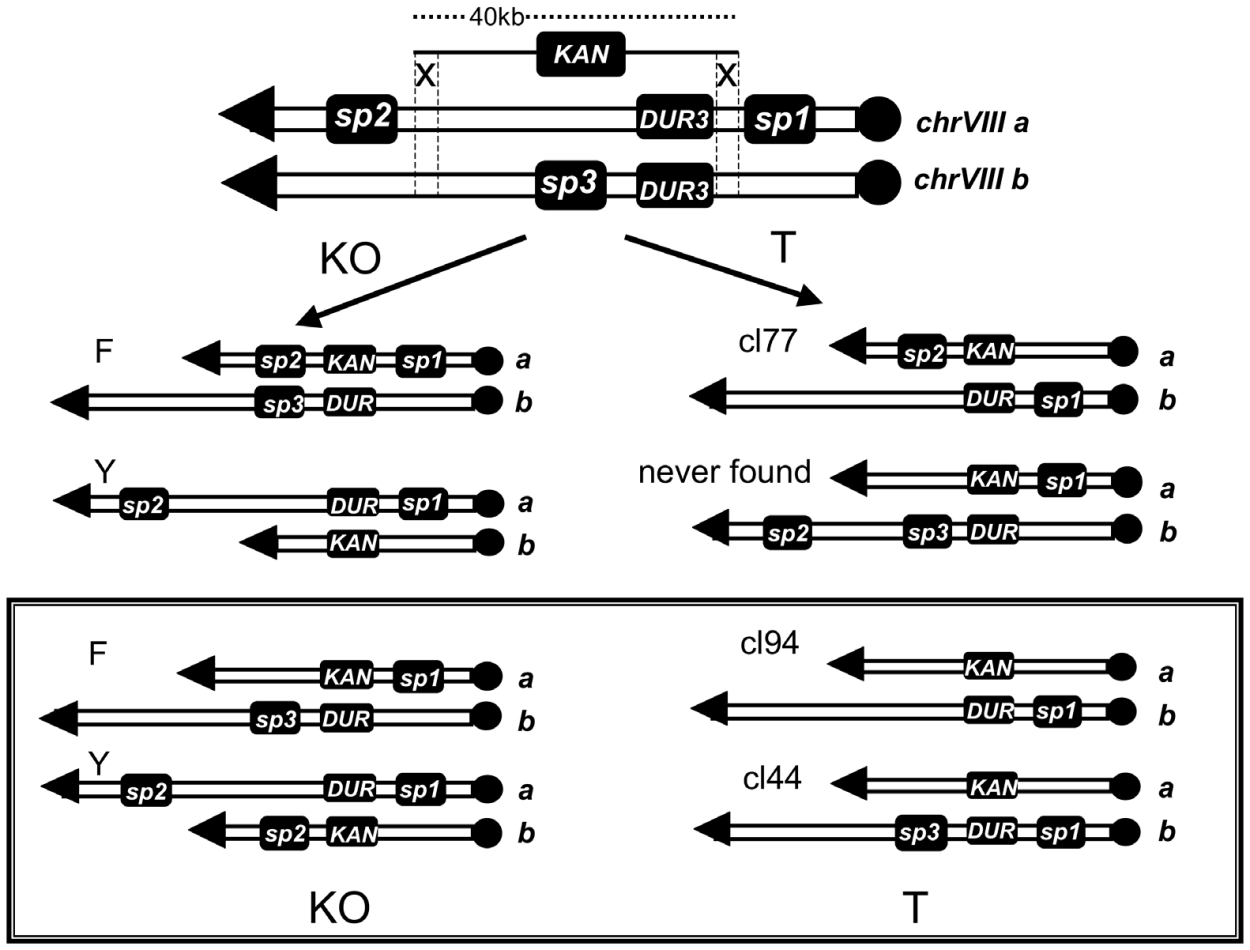
KO and translocation (T) events with short and long homologies between two homologous chromosomes VIII. The two homologs are labeled here as chrVIII **a** and **b**, and are bridged by a DNA cassette carrying kanamycin resistance (KAN). On the top, the molecular event that can give rise to KO (left) and to T (right) as previously reported [9] using a cassette with short homologies (40 nt). F and Y are the two possible chromosome configurations for the KO as reported in the text. The only translocant found was cl77 while the opposite configuration was never detected. At the bottom (framed area), the different result obtained with a homology of 400 nt (reported in this work). On the left the transformants with the KO in the homologous chrVIII **a** (F); it is shown that spacer sp2 is lost from both chromosomes VIII; on the contrary, in the transformants where the KO happened in the homolog chrVIII **b** (Y) sp2 is present on both chromosomes VIII. This LOH, with the absence of sp2 on both chromosomes, is maintained also in the two translocants cl94 and cl44 (frame, right) while cl44 only, which is in *rdh54Δ*/*rdh54Δ* background, has retained also sp3 as it is shown experimentally in details in Fig. 6C.

In the absence of *RDH54* (Fig. 1), only a slight increase of BIT efficiency was detected, in spite of the occurrence of spontaneous translocation event between LTRs on different chromosomes, as just described (*see characterization of the translocants*). Since it is well known that *RDH54* is mainly involved in mitotic diploid-specific recombination [39], we decided to verify if it had any role in BIT between homologous chromosomes. To obtain a significant translocation frequency, the two DNA homologies have to be located at a distance of at least 40 kb from each other as previously reported [9], otherwise KO would be highly preferred. We built a San1-derivative diploid strain, named YF123, in which the two pairs of chromosome VIII are labeled with three artificial marker sequences of 125 nt each (spacers), placed upstream (sp1) and downstream (sp3) the *DUR3* gene and upstream (sp2) the gene *SBP1* that is located at 40 kb from *DUR3* [9]. Using the diploid strain YF123 wild type and the YF123 *rdh54Δ/rdh54Δ*, we repeated the BIT extending the homology up to 400 nt, thus probing at the same time whether the length of the homology, together with the absence of Rdh54, may affect translocation efficiency between homologs. The experiment and the results are summarized in Fig. 5, where the various configurations that could be adopted by each chromosome are indicated with the letters F, Y and T. The presence of the spacers allowed to discriminate by colony-PCR between the two homologous chromosomes VIII and therefore between the possible recombination outcomes [9]. In fact, the size of the PCR products is different with or without the spacer and lets to understand on which of the two chromosomes VIII the integration took place for each event (Fig. 5). More than 300 transformants were collected and, among them, two were verified translocants between the two homologous chromosomes VIII, one in the wild type (cl94) and one in the *RDH54* deletant (cl44). More in details, of the 154 clones obtained in the wild type, 24 were in chromosome configuration F, 11 in Y, 1 in T (named cl94, while the opposite configuration was never found) and 118 were ectopic. Comparing these data with those obtained with shorter homologies [9], one could conclude that, despite the ten-fold increase of the homology region, the efficiency of the KO has only slightly improved (from 11 to 16% in configuration F and from 3 to 7% in configuration Y, Fig. 5) while the efficiency of the specific translocation remains approximately around 1%.

**Figure 6:**
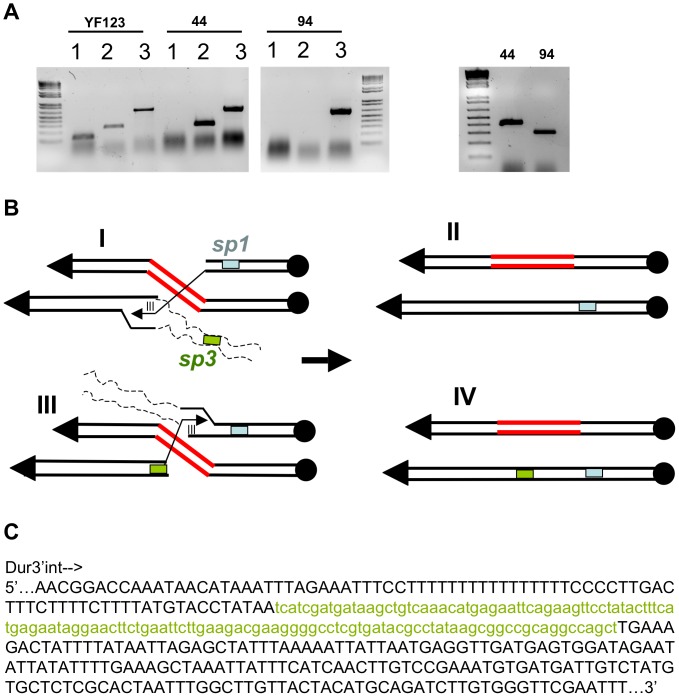
Characterization of the translocants between homologs and proposed model explaining the role of Rdh54. A) Characterization through colony-PCR of cl44 and cl94. The presence of spacer2 (lane 1 = primers sp2/sp2Fw), spacer3 (lane 2 = primers sp3/Dur3’int) and spacer1 (lane3 = primers Sp1/Dur3R) are shown from left to right in the wild type strain YF123, in the translocant 44 (*rdh54Δ*/*rdh54Δ* background) and in the translocant 94 (wt YF123) respectively. In the panel on the right the amplification size of the region surrounding spacer3 in clone 44 and 94 is compared (DurEXT/Dur3’int). The partial sequence of these fragments is shown in details on panel C. B) Two hypothetical models to explain the loss or the retaining of spacer sp3 in the wild type cl 94 (I, II) and in the *rdh54Δ*/*rdh54Δ*cl 44 respectively (III, IV). C) DNA consensus from the alignment of a partial sequence of cl44 and cl94 around spacer3 confirming that only in the first one the spacer is present (here indicated as green, small letters). The capital letters indicate the nucleotides present in both clones.

While the length of the homologies did not raise the low frequency of translocation between homologs, it seems, vice-versa, to affect the molecular mechanism of the event. Indeed, in the translocants and in the KO-transformants analyzed, the presence or the absence of the distal spacer sp2 on both chromosomes testified a loss of heterozygosity (LOH) due probably to a gene conversion (GC) followed by crossing over (CO) (Fig. 5, in the frame). This LOH phenotype was never observed in previous experiments using a short homology (40–60 nt) where only the expected event of GC was found [9]. Rdh54 does not take part in the molecular pathway leading to the CO since the same LOH, with the absence of the distal sp2 on both chromosomes, was observed in the translocant *rdh54Δ/rdh54Δ* (cl44) (Fig. 5). Moreover, Rdh54 does not have any influence on the integration frequency related to the extension of the homology. In fact, similarly to the wild type, in *rdh54Δ/rdh54Δ* background, among 157 transformants 15 were F, 11 were Y, 1 was T (cl44), and 130 were ectopic. Nevertheless, it seems that Rdh54 may affect the Holliday junction (HJ) resolution point following the integration. Indeed, in translocants cl77 and cl94, obtained in the wild type with short and long DNA homology, respectively, spacer sp3 is lost, but it is still present in the translocant cl44 in the absence of Rdh54 function (Fig. 5). Chromosomal configuration is exactly the same in the three translocants (integration upstream kanamycin in chromosome **b** followed by integration downstream kanamycin in chromosome **a**, see Fig. 5), denoting also a preferential directionality for the translocation. However, sp3 is present only when Rdh54 is absent (Fig. 6A). Therefore, the lack of Rdh54, coupled with BIT between homologs, might affect the feature of the resultant translocant, but it is surely not involved in the LOH phenomenon induced by the extension of the homologies. The same model of invasion, chromosomal resection and resolution previously proposed to explain the translocation in cl77 [9] may explain also the generation of cl94 in this work (Fig. 6B) with the only difference that a phenomenon of CO due to the increase of the homology may be responsible for the loss of sp2. In the wild type (Fig. 6B, panels I and II), after the integration of the kanamycin cassette and the consequent loss of 40 kb (dashed lines), the free end of the broken chromosome VIII invades the homologue (I); the rescission of 40 kb brings to the loss of the spacer3 (II). When *RDH54* is missing (Fig. 6B, panels III and IV) the other free end of chromosome VIII, holding sp3, invades its own homologue (III) leading to a different resolution product (IV) and suggesting an active role of Rdh54 in processing the HJ. In conclusion, the presence of the spacer sp3 in absence of Rdh54 may indicate a putative role for Rdh54 in driving the process of migration and resolution of the HJ at different points. Of course, the collection and analysis of an increased number of translocants in absence of Rdh54 could validate this hypothesis conferring it a stronger statistical meaning.

The presence of the spacers in YF123 *rdh54Δ/rdh54Δ* allowed us to verify that BIR is effectively responsible for a partial trisomy during BIT between heterologous chromosomes. In fact, from the analysis of the six *rdh54Δ/rdh54Δ* translocants, we detected by PCR the presence of the two original chromosomes and of the new one harboring kanamycin (data not shown). We found that the homologous chromosome **a** (as illustrated in Fig. 5) was copied in five of them while the homologous **b** was copied in cl23 only. The type of BIR involved in these BIT events is discussed in the next section.

### Pol32-independent BIR is Required to Complete the Bridge

We previously demonstrated [10] that BIT may be completed through Break-Induced Replication (BIR) and we proposed that the efficiency of the repair is reversely proportional to the distance of the sequence homology from the telomeres.

In this work, we collected 48 G418-resistant-transformants in *pol32Δ/pol32Δ* background, performing 28 different transformations (Table S2, Fig. 1). These data indicate a transformation level of *pol32Δ/pol32Δ* with a linear DNA cassette, that is seven-fold lower than in the wild type, while the transformability with a circular plasmid is only half (Table S1). Probably, the absence of Pol32 function does not allow an efficient repair of the DSB generated within chromosome XV after the integration of the first end of the cassette, and this absence of BIR repair leads to death of the majority of the cells because of the persistence of an unrepaired DSB. However, recovery of specific translocants is still possible, also in absence of Pol32 and, paradoxically, the translocants account even for the 20% of the total integration events. This could happen because the events of break repair through BIR, exploiting a micro-homology, are strongly impaired in absence of Pol32. Nevertheless, considering the reduced overall number of events in the mutant (Fig. 1), Pol32 is definitely responsible for a certain number of BIT translocations in wild type cells. From ongoing studies we can predict that its importance in the BIT process seems to be limited to the S phase of the cell cycle (Tosato and Rossi, personal communication).

**Figure 7:**
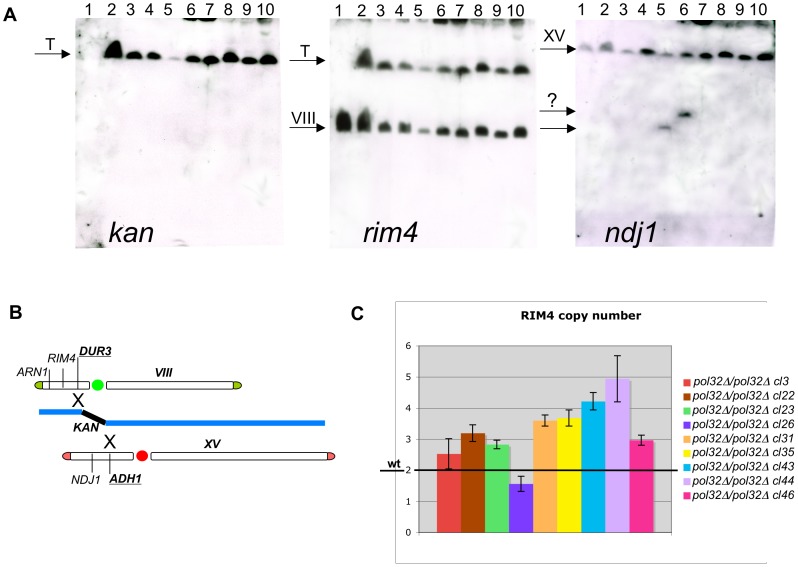
Characterization of nine independent *pol32*Δ/*pol32*Δ translocants. A) Southern hybridization of genomic DNA from all the *pol32Δ*/*pol32Δ* translocants with probes against kanamycin, *rim4* (chromosome VIII) and *ndj1* (chromosome XV) *loci* are shown. Lane 1:wild type San1, 2: *pol32Δ*/*pol32Δ*cl3, 3: *pol32Δ*/*pol32Δ*cl22, 4: *pol32Δ*/*pol32Δ*cl23, 5: *pol32Δ*/*pol32Δ*cl26, 6 *pol32Δ*/*pol32Δ*cl31, 7: *pol32Δ*/*pol32Δ*cl35, 8 *pol32Δ*/*pol32Δ*cl43, 9 *pol32Δ*/*pol32Δ*cl44, 10: *pol32Δ*/*pol32Δ*cl46. T indicates the band of the translocated chromosome. The question mark indicates unknown bands coming from a rearrangement of the left part of chromosome XV on the membrane hybridized with *ndj1* B) Scheme of the location of the probes used with respect to the breakpoints (underlined) C) The *RIM4* copy number is reported with its own legend on the right. The results are referred to the wild type (here represented by a line with a value of two); each bar is the result of nine independent readings (see Materials & Methods for details).

All the *pol32Δ/pol32Δ* translocants were analyzed in details by Southern blot, sequencing of the junctions and microscope staining, revealing the expected patterns of hybridization (Fig. 7A), stability of the translocated chromosome, absence of base-pairs errors and absence of phenotypic defects (with the exception of cl26, Fig. S6B). The phenotypic defects and the strong mortality of cl26 may be partially attributed to the rearrangement of the fragment of chromosome XV generated by the translocation (Fig. 7A, probe *ndj1*, lane 5). The actual causes of mortality are presently under investigation to understand if these translocants simply fail to grow, arrest in G2 or undergo a mitotic catastrophe.

To verify whether a partial trisomy of chromosome VIII is present also in *pol32Δ/pol32Δ* translocants, justifying a BIR-like mechanism, densitometric readings of semi-quantitative-PCRs were performed (Fig. 7C). Almost the majority of the translocants showed three copies of the *RIM4* gene that is located on the region between the telomere and the *dur3* point of translocation. At least two of them (cl43 and cl44), revealed further copies of this region. Cl3 and cl26 gave the same result of the wild type. Cl26 showed high instability of the translocated chromosome (about 30% of chromosome loss was verified by replicas on G418, data not shown). Therefore, we can hypothesize that a replication event from *dur3* toward the left telomere of chromosome VIII is probably responsible for the formation of the translocants also in absence of Pol32. To understand whether the mechanism that leads to the translocation via BIT, in absence of Pol32, is the half-crossover model [40], it would be necessary to assess if the increased copy number of *rim4* in many translocants (Fig. 7) is due to endo-reduplication of the translocated chromosome or of the endogenous chromosome VIII. However, since in the half crossover model the template chromosome is always lost and the chromosomal fragment (CF) is never duplicated [40], it is unlikely (Fig. 7B, C) that this model may justify translocants formation via BIT at least in the majority of *pol32Δ/pol32Δ* BIT translocants.

## Discussion

The efficiency of the bridge-induced translocation (BIT) system [8] depends on the recombinagenicity of the selected *loci*, on the length of the homology and on the genetic background of the yeast strain. In fact, it has been demonstrated that, i) hyper-recombination *loci,* such as pre-telomeric regions, are BIT hotspots [10], ii) that the extension of the homology from 40 to 65 nucleotides results in the doubling of the efficiency of translocations for the same *loci* [8], and, iii) that the rDNA-repeated region within Chromosome XII, whose size varies from strain to strain, strongly affects the final genomic outcome by attracting variable proportions of integration of the DNA bridge molecule [8]. Regardless of the different chromosomal configuration, the resulting translocants are the likely products of a two-step mechanism, relying on the HRS for the first integration event and on HRS or BIR for the second integration and completion of the bridge, respectively. To better understand the molecular mechanism underlying BIT and to find out the genetic determinants involved, we analyzed the effect of bridge-induced translocation in several yeast strain mutated in recombination-specific genes, focusing on the *RAD54/RDH54* pair as members of the HRS and on *POL32* as BIR-specific gene. We showed that transformation with a BIT cassette harboring two homologies for two distinct chromosomes of yeast generates an ensemble of events that are completely different from those observed in a GT experiment and probably are due to activation of different molecular pathways.

We found that when *RAD54* is deleted, BIT events are repressed, but the possibility to obtain non-reciprocal translocations is completely rescued restoring the Rad54 function. Rad54 is a member of the SWI/SNF-chromatin remodeling complex and of the Rad52-epistasis group in which it is essential for the turnover of Rad51 at the DSBs and, moreover, it is responsible for the transition from homologous pairing to DNA synthesis [41]. This Rad54 peculiarity may also explain the almost complete absence of synthesis at *dur3 locus* in *rad54Δ/rad54* transformants after BIT, which justifies the absence of translocants and of one-side “integrants”. Several *dur3* “integrants” were in fact previously identified as self-replicating-copy fragments of variable size carrying an origin of replication [8]. Furthermore, it has been recently demonstrated that Rad54, differently from other universal HR factors such as Rad51, is specifically involved in GT or intra/inter-chromosomal association and promotes HR between chromosomes and extra-genomic DNA in human sarcoma cell lines [42]. It is clear that since Rad54 mainly works on the higher structure of chromosomes, its absence implies not only a decrease in GT, as we confirmed in this work, but also the reduction of interaction between the BIT artificial, exogenous cassette and the genome. Vice-versa, we demonstrated that the over-expression of *RAD54* still allows the recovery of translocants via BIT although with lower percentages respect to the wild type. Indeed, it was previously reported that an over-expression of *RAD54* unexpectedly leads to a decrease in conversion tract lengths [43], and our data confirm that the stoichiometric balance of Rad54 is essential for an efficient post-synaptic phase of the HR between exogenous linear DNA and the yeast genome. In addition, we verified that, when *RAD54* is over-expressed, after the first translocation event secondary LTRs-mediated rearrangements could occur. It was recently demonstrated [44] that non-allelic homologous recombination (NAHR) might arise because DSBs activate Ty recipients that are distal up to 50 kb from the break site and that may recombine with non-allelic Ty donors. This phenomenon, known as break distal recombination (BDR), can take place spontaneously, though infrequently, in yeast cells, accounting for chromosomal number variations. When the DSB occurs inside a unique region, the BDR is preferred rather than single strand annealing (SSA) or GC to repair the break and, after an extensive DNA resection up to the nearest LTR, it generates severe GCRs [40]. It is inferred from our data that this pathway is strongly elicited when *RAD54* is over-expressed. Therefore, we can propose that, after the first, induced BIT translocation event, the over-expression of *RAD54* promotes further recombination events between repetitive regions next to DSBs. The DSBs triggering BDR in our translocants are either artificially induced by BIT (such as the break next to *adh1* within chromosome XV) or spontaneously arisen (such as the one within the pre-telomeric sequence of chromosome VIII and the ones within the rDNA region of chromosome XII). We identified the break-distal LTRs that recombined in the translocants over-expressing *RAD54*. In effect, one of the implications of the BDR is that the recombinant junction never coincides with the site of the initial lesion. It is quite obvious that, since the *LTRYHRCΔ16* triggers BDR in all our translocants, a fragile site that predisposes to recurrent instability exists somewhere next to this region, inside the right telomere of chromosome VIII. On the other hand, the mechanism to maintain the rDNA copy number generates continuous DSBs, rendering this arm of chromosome XII one of the most fragile sites of the yeast genome [45] and justifying secondary rearrangements via BDR distal to this region in our OeRAD54 clones.

Many chromatin remodelers have been correlated with ploidy maintenance [46] and, among them, *RAD54* in particular is found over-expressed up to 5-fold in prostatic cancer cells characterized by recurrent non-reciprocal translocations [21], [47]. In these translocations, as in our clones, there is a loss of chromosome material next to the joining point, with consequent LOH. Moreover, the chromosomal fragile sites triggering translocations in prostatic cancers cells are often characterized by sequence repeats such as LTRs of endogenous retroviruses and small unstable poly-dispersed circular DNAs (spcDNAs) [48]. These observations may lead us to hypothesize that a BDR mechanism similar to the one observed in our OeRAD54 translocants is responsible for recurrent aneuploidy-associated translocations found in these types of tumors.

While *RAD54* is important for the regulation of BIT between heterologous chromosomes, we found that the diploid-specific *RDH54* (*RAD54B* in mammals) seems to affect HJ resolution mechanism between homologous chromosomes, although a greater number of translocants is required to confer statistical meaning to the data. It was already known that Rdh54 is necessary for inter-homologues, but not intra-chromosomal gene conversion [39] and that it is implicated in the meiotic co-localization of Rad51 and Dmc1, D-loop formation, and establishment of crossover interference [49], [50]. Like Rad54, also Rdh54 promotes branch migration and unwinds three-strand DNA structures [51]. In our experiments, the presence of spacer sp3 on the translocant between the two homologous chromosomes VIII in *RDH54* deletants, predicts for Rdh54 an unforeseen role in determining the migration direction of recombination intermediates. Its absence heavily affects the genomic outcome of the translocation and it probably engenders the requirement of other factors (Rad54, Rad51) in a complex multi-interactive model of HJ resolution. These data are in agreement with the idea that Rad54/Rdh54 can promote four-strand branch migration in either the 3′ → 5′ or the 5′ → 3′ direction relative to the displaced ssDNA strand and that the defects and changes in assembly of Rdh54 around the HJ could effect its bi-directional translocation on DNA [18]. Finally, the data concerning the instability of the translocants in heterologous translocations indicate that the well-known meiotic ATPase Rdh54 [52] might be involved as well in mitotic pathways of chromosomes pairing and it might play a fundamental role in chromosomal mitotic segregation of diploid cells. In effect, there are indications that the loss of an endogenous chromosome in a *rdh54Δ/rdh54Δ* diploid strain is two-fold greater as compared to the wt [53]. Experiments are ongoing in our lab to verify whether the loss rate of a translocated, hybrid chromosome is comparable to the one of an endogenous chromosome and how much this rate may vary testing translocants of different chromosomes and different lengths in *rdh54Δ/rdh54Δ* strains (Tosato and Sims, personal comm.).

While Rad54 and Rdh54 seem to be so fundamental in the regulation of BIT events, Pol32 is a dispensable factor. Even if it was demonstrated to be essential for ectopic BIR induced by HO-mediated DSB [27], its loss does not prevent the formation of stable BIT translocants via HRS. However, we found in this work that Pol32 favors ectopic translocation events, based on micro-homology recombination [8], allowing a higher survival rate after BIT-triggered chromosomal DSB. It is true that the relative translocation in absence of Pol32 is three-fold higher than the wt (Table S2a), but the number of treated cells to get the same amount of transformants is nine-fold higher. Therefore, Pol32 does not repress translocations, but promotes mainly the micro-homology-mediated healing outcomes (such as ectopic integrations and one-end integrations). By our recent experiments (Tosato, Rossi and Bruschi, unpublished results) it seems that the only participation of Pol32 in the translocation via BIT is limited to the S-phase where, probably, there are some translocations events due to Pol32-dependent BIT. We therefore postulate that the translocants that we recovered without Pol32 are generated in other phases of the cell cycle and are due to other genetic players that should be identified. Therefore, we conclude that the BIR pathway responsible to complete the bridge and to produce aneuploidies in BIT has a different genetic requirement than the HO-induced BIR, which is characterized by breaks with homeologous or heterologous ends. This conclusion is perfectly in agreement with the recent observations that many HR-GCRs are Pol32-independent and that Pol32 is not required for allelic BIR [54]. Moreover, our data suggest that this HR-induced BIR is suppressed in absence of Sgs1. Despite the fact that the 25% of the transformants in *sgs1Δ/sgs1Δ* mutant were *adh1*-integrants and that ectopic integrants (among them, many ectopic translocants) rose up to 75% (Fig. 1, Table S2a), the targeted translocation efficiency was dropped by BIR failure at *dur3 locus*. Again, vice-versa, Sgs1 promotes HO-induced BIR [27], confirming the hypothesis that the homology at the ends of the break drives the choice of the molecular players in DSB-induced replicative repair. The unexpected high rate of ectopic integrations in *xrs2Δ/xrs2Δ* either with BIT or with a GT cassette are apparently in contrast with a described decrease in illegitimate recombination in *rad50* and *xrs2* mutants. [55]. But there are two main differences between our data and previous reports: the first is that BIT and GT were tested here in diploid cells while in previous works only haploids were used. Diploids not only allow a higher statistical rescue of survivors after the first DSB, but also display a completely different choice of utilization of the HR genetic pathway with predominance of HR against NHEJ. Secondly, in our experiments only DNA cassettes with perfect homology to their chromosomal site were utilized. Both in BIT and in GT experiments, when *XRS2* is missing, there is the prevalence of integration at one side only. In BIT, the integration events occur prevalently in *adh1* only (66%, Table S2a, Fig. 1) while in GT, 58% of the events are consequences of one-homologous-end integration only (data not shown). These results suggest that Xrs2 in yeast is not very important for HR, as also reported in other organisms [56] and that when this protein is missing during BIT the cell may exploit the HR to integrate one end of the cassette while the genomic instability, due to the mutation, will help in integrating the second end. If the break is generated in a haploid cell with a fragment without homology [55], since Xrs2 is involved in illegitimate integration, the obvious conclusion will be the death of the haploid cell (as found in [55]). A systematic analysis of the numerous putative ectopic transformants of *xrs2Δ/xrs2Δ* and *sgs1Δ/sgs1Δ* is currently under investigation to understand if they are ectopic translocants due to a micro-homology recombination or simply the consequence of a random capturing of the DNA cassette by recombination hotspots.

We therefore conclude that BIT is strongly subjected to Rad54 in terms of efficiency and final genomic outcome and that an extensive break-induced synthesis event, which is homology-dependent and Pol32-independent, is bound to complete the translocation bridge. Moreover, in the context of the proposed two-steps model for BIT, Xrs2 is important for the second DNA-end integration and for ploidy stability of the translocants (see lane 14, hal9 Fig. 2C), as well as Rdh54 (Fig. 4). Rad54 is fundamental for the first step of the model while Top1 and Elg1 are dispensable and Msh2 seems not affect homologous ends integration. Further elucidations on the specific role of other factors such as Sgs1 and Xrs2, which are here anticipated as affecting the phenomenon, are under investigation via a thorough analysis of BIT-induced replication in S-phase-synchronized yeast cells.

## Materials and Methods

### Yeast Strains and Media

The diploid *S. cerevisiae* strain San1, constructed in our laboratory [57], [58], was used to induce the translocation between chromosome XV and VIII and as control strain throughout this work. Strain YF123 is a derivative of San1, specifically modified to work with translocations between the two homologous chromosomes VIII that have been differentially labeled with unique spacers for their identification [9]. The double deletants of the genes *MSH2, SGS1, XRS2, RAD54, POL32, ELG1, TOP1* (in the San1 background*)* and of *RDH54* (YF123 background) were all obtained following a gene disruption methodology for both alleles based on the 2-micron recycling system [59].

YPD (Difco) supplemented with geneticin G418 (final conc. 200 µg/ml, Gibco) was used as selective medium. For the double selection –URA +G418, SE drop-out-media (with ammonium glutamate instead than ammonium sulfate) were prepared as previously described [60]. For the selection of the translocants complementing and over-expressing *RAD54*, an additional replica plating was necessary in order to eliminate the usual background present in SE –URA +G418 medium.

### Molecular Biology Techniques and Microscopy

Genomic DNA extraction was performed using an optimization of the total DNA extraction protocol [61] reported in Methods in Yeast Genetics [62]. The RNA extraction to test the expression level of CRAD54 and OeRAD54 strains was performed with RNeasy Mini Kit (Qiagen) when culture density was within 1.5–2.0×10^7^ cells/ml. Amplification of the DNA bridge cassette between *adh1* and *dur3 loci* and PCR confirmation of the transformants were performed according to [8], [9]. The PCR products were obtained by HF-Taq and subsequently quantified. Cells were counted and transformed while in exponential log phase (1.5–2×10^7^/ml) and G418 resistant transformants were obtained using the lithium-acetate transformation for the PCR-based gene replacement method [63]. Confirmation of *DUR3* KO transformants was performed by colony-PCR following the standard procedures recommended in the guidelines for EUROFAN B0 Yeast Program (Wach, Brachat and Philippsen, 1996). A scheme for the screening of the positive transformants is reported in Fig. S1. All the translocants were confirmed by Southern hybridization, by PCR on genomic DNA and by DNA sequencing. Chromosome separation and Southern hybridizations were performed as previously reported [8]. The primers used to amplify the probes are listed in the Table S3. The amplification of the 6.5-kb gap between the LTRs YHRCδ16 and YOLCδ3 in the over-expressing *RAD54* (OeRAD54*)* clone 38 (cl38) was performed using the Extender System Kit (5 Prime) following the instructions of the supplier. Microscope procedures and equipment were previously described [9] and the FUN staining (Molecular Probes) was performed according to [10] using a final concentration for the dye of 6 µM.

### Expression Vectors and Artificial Constructs

Complementation and over-expression of *RAD54* were carried out after amplification of the gene with its own promoter and terminator (Pwo SuperYield DNA Polymerase, Roche) and its cloning and sequencing in the bacterial vector pMOSBlue (GE Healthcare). The verified construct was then cut and re-cloned in the single copy, centromeric vector PRS416 [64] and in the multi-copy vector PRS426 [65] for the complementation and over-expression, respectively. Semi-quantitative RT-PCR (Fig. S2) and DNA copy-number (Fig. 4, 7, S4) determination were performed as described previously [10]. Each result was the average of nine independent readings and it is represented as a bar in the figures.

To extend the homologies of the cassette used for translocations between homologous chromosomes up to 400 nt, two fragments of 382 nt and 431 nt, located 40 kb from each other within chromosome VIII, were amplified by high fidelity Taq DNA polymerase and cloned in a pFa6AKANMX4 plasmid cut by PvuII/BamHI and EcoRI/EcoRV. This construct was then used as template to amplify the BIT cassette. All the primers chosen for the generation of the cassette with 400 nt of homology and their precise locations in the genome are reported in the Table S3.

### BIT and GT Efficiency Computation

The same amount of linear DNA (10 µg) was used in all the transformation experiments with the BIT cassette while 400 ng of plasmid YCp50 were used to test the transformability of the wild type strain and of the set of deletants (reported in Table S1). Therefore, since the amount of DNA was always the same in each transformation, we considered frequency (**ν**) as an approximation of efficiency ( = **ν**/[DNA]). This approximation was applied also to GT experiments, where 5 µg were always used per each transformation. The only variables are the number of events (one-side integration, ectopic integration, two-side integration, - i.e. translocation) and the number of treated cells. The total amount of cells per transformation was between 1.18 and 1.85×10^8^ for GT experiments and between 2.2 and 3.4×10^8^ for BIT experiments (see Tables S1 and S2b). The term “ectopics” refers to randomly integrated transformants, regardless of the homology; *adh1-int* or *dur3-int* refer to transformants obtained with a cassette correctly integrated at one side only (*adh1* or *dur3* respectively) and ectopically integrated on the other side. AD Translocants (**Tsl**) are transformants obtained by correct integration of the cassette at both *adh1* and *dur3 loci*. The transformation frequency (**νt**), as also the frequency of integration in adh-only (**νiadh**), in dur3-only (**νidur3**), of ectopic integrations (**νiect**) and of translocation (**νTsl**) respect to each strain transformability (**νp**) are summarized in Table S2b. These values were obtained dividing the number of transformants collected on G418 per each event (Table S2a) by the total number of cells used (Table S2a). An example of computation is reported as follows:

San1 (wt) transformation frequency (**νt**) = total number transformants/average number cells in each transformation **x** total number transformations = 51/3.46×10^8^×3 = 4.91×10^−8^. This value is then divided by the transformability of the strain tested with YCp50 (**νp**). **νt**/**νp** = 4.91×10^−8^/9.4×10^−5^ = 0.52×10^−3^ that is the number reported in Table S2b and that was used to draw Fig. 1. This procedure was applied to the whole set of deletants and it was repeated not only for the **νt**, but also for **νiadh**, **νidur**, **νiect**, e **νTsl** (nomenclature, values and standard error of the mean in Table S2b). The raw data concerning number of transformations, number of transformants, and number of treated cells are reported in Table S2a. The **νp** values are in Table S1.

The same procedure was also applied to calculate the frequency of transformation and of KO with the *ΔDUR3* cassette as summarized in Supplementary Fig. S1. The frequency of transformation **νt** and of real disruptions (**νko**) in the deletants and in the wild type, divided by the transformability with YCp50 (**νp**) and the number of treated cells are all reported in Table S1. All the data enclosed in Tables S1 and S2 were analyzed for statistical significance. The *P* value for the GT (Table S1) is ≤0.05 for all the mutants with respect to the wt with the exception of *pol32Δ/pol32Δ.* For this mutant the number of collected transformants was considered reasonable on the basis of a previous study [27] that demonstrates the involvement of Pol32 as limited to the BIR pathway only. For *rad54Δ/rad54Δ* and for *rdh54Δ/rdh54Δ* the P values are in the order of 0.05, but our observations of a decreased GT are strongly supported by similar previous results obtained in independent experiments [25], [66].

The P value was calculated for all the outcomes (one-side integration, ectopic integration, two-side integration, - i.e. translocation) plotted in Fig. 1 using the data in Table S2. For the mutants of the genes *RAD54, SGS1, POL32* that are showing a striking difference in BIT efficiencies with the wild type - and were therefore further analyzed in this work- P is always <0.05. For the mutants giving outcomes very similar to the wild type (i.e. translocation in *top1Δ/top1Δ*) P is higher than 0.05 (0.05≤P<0.1) and more transformants should be collected to increase the statistical significance. Since this was not the purpose of this work, these outcomes were not analyzed in details or discussed.

## Supporting Information

Figure S1 **Histograms representing different transformation and knock out frequencies with a GT cassette.** Frequency of transformation **νt** (left, dark bar) and of knock out **νko** (right, light bar) of the gene *DUR3* in the wt and in eight different mutants divided by the transformability of each strain *(* ***ν*** **p**). **νf** (f = fragment) in the y-axis indicates **νt** or **νko** for the left and the right bar respectively. Computational data used to draw the histogram are reported in Table S1. At the bottom, a scheme of the knock out with the relative homologies (H = 65 nt) is shown. The arrows indicate the orientation of the gene transcription. The homology H1 was also used with the BIT cassette (Figure 1). The primers (k1, D–F, D–f; k2, D–R, D–r) used for the verification of the integration by colony-PCR are also reported (for the primer sequences see Table S3). In the frame an example of one-end integration (a), two-end integration = KO (b) and ectopic integration (c) is illustrated. Ectopic integrations, regardless the homology, might occur also following integrations of the two ends into two different chromosomes (they generate in this case ectopic translocants). H1 and H2 in ectopic integrations and in one-end integrations may be completely or partially lost (degraded) as we previously reported [8].
(PDF)

Figure S2 **RT-PCR analysis of** ***RAD54*** **expression in the complementing (CRAD54) and over-expressing (OeRAD54) strain with the distribution of BIT integration events.** The primers used to test *RAD54* expression are listed in Table S3. A) Lane 1: wild type San1, 2: *rad54Δ/rad54Δ*, 3: CRAD54, 4: OeRAD54, 5: PCR negative control. *HSC82* is a gene constitutively expressed, used as expression reference. On the right, the semi-quantitative RT-PCR analysis is quantified by laser-scanning densitometry as described in the Materials & Methods section. The results are plotted as histogram bars relative to two copies of *RAD54* present in the diploid wild type on lane1. B) Number of transformants, translocants and one-end integrants (either in the *adh* or in the *dur locus*), in CRAD54, OeRAD54, the heterozygous strain (*RAD54/rad54Δ*) and in the wild type strain San1.
(PDF)

Figure S3 **Genetic and phenotypic characterization of OeRAD54cl37.** The first line on the top represents the consensus among the three OeRAD54 translocants and one translocant complementing *RAD54* (CRAD54cl1) used as control. The 65 nt-homology used in the BIT event (H1 in the scheme of Figure 1) is underlined in yellow. The dots indicate the duplication (which is present only in OeRAD54cl37). A segment of 40 nt is outlined in orange and another one, consisting of 30 nt, is squared in green to highlight the origin of the duplication. One T (underlined in pink) was added by the cell within the two short duplicated segments. On the bottom of the panel, the phenotype of OeRAD54cl37 is shown: A) picture without staining evidencing the presence of dead ghost cells; B) DAPI staining showing nuclear fragmentation; C) calcofluor staining reveals unusual cellular shapes; D) FUN combined with calcofluor staining confirms the high cell mortality.
(PDF)

Figure S4 **Analysis of the junction between chromosomes VIII and IV in** ***rdh54*** **Δ/** ***rdh54*** **Δ.** A) Scheme of the regions involved in the rearrangement; the resulting chromosome is drawn as filled color. Two asterisks indicate the breakpoints; the primers, used to amplify the region (DCD1-rev and YDR262-rev), are also reported. The *loci rim4* and *cdc23* are indicated in the upper scheme (chromosome VIII); the *locus act1* (chromosome VI) was used as internal control. The junction between chromosomes VIII and IV was successfully amplified using the primers REV DCD1 and REV YDR262w (Table S3) and sequenced (B). It revealed a complex rearrangement in *rdh54Δ/rdh54Δ* background involving two LTRs sharing strong similarity (C). On chromosome VIII the break is within the LTRΔ10 while on chromosome IV it takes place within LTRΔ21. B) The sequencing electropherogram of the junction IV/VIII is reported in details. The switching between the two chromosomes is highlighted. C) BLAST (Basic Local Alignment Search Tool) of the two LTRs where the translocation took place. The query corresponds to a sequence region of chromosome VIII within LTRΔ10 (SGD coordinates: 389177–389400) and the subject corresponds to a region of chromosome IV of the LTRΔ21 (SGD coordinates: 987150–987380). The alignment allows the visualization of the minor differences between the two LTRs and it allows the comparison (in yellow) with the real, hybrid sequence (B) at the point of the junction.
(PDF)

Figure S5 **Southern hybridization analysis of the OeRAD54 translocant bridging chromosomes XVI and IX (SUSU).** DNA hybridization with probes against the *loci arn1, gal4*, *glc1*, *kan* and *dal4 (*for primers see ref. 10) to verify the presence of both arms of the chromosomes. Lane 1: wild type San1; 2: AD translocant (XV–VIII) (used as hybridization control); 3: cl48 SUSUOeRAD54 (translocation IX–XVI). On the left, a lane of CHEF chromosomes separation indicates the chromosomes probed. The *ARN1* gene is located on the left arm of chromosome VIII next to *RIM4*. The location of the other probes is indicated in the scheme below the hybridization panels.
(PDF)

Figure S6 **DAPI and FUN staining of several translocants showing peculiar phenotypes.** A) Translocants number 44 and 46 in *pol32Δ/pol32Δ* without (left) and with (right) fluorescence microscopy after DAPI staining; single cells with two nuclei are visible. B) *pol32Δ/pol32Δ* cl26 is characterized by strong karyokynetic defects such as germination tube formation and by a strong flocculation. C) FUN staining of four translocants characterized by point mutations at the breakpoints; from left to right: *rdh54Δ/rdh54Δ*cl30, *top1Δ/top1Δ*cl18, *elg1Δ/elg1Δ*cl14, *xrs2Δ/xrs2Δ*cl6. Dead cells appear as yellow-colored. Point mutations, insertions or deletions around the breakpoints were never detected in the three translocants obtained in the wild type background.
(PDF)

Table S1 **Frequency of strain transformability with the tester plasmid Ycp50 (νp), frequency of strain transformation (νt) and of strain knock out (νko) with the linear cassette.** The first column contains the number of transformants T obtained on -URA with YCp50 in each strain using always 1×10^7^ cells. The second column contains the frequency of strain transformability (**νp**) calculated as the number of transformants on –URA divided by the number of treated cells. The third column summarizes the number of transformants (**T**) obtained on G418 with the linear cassette. The fourth the number of real disruptants (**T KO**) with the linear cassette. The frequencies of transformation (**νt**) and of knock out (**νko**) are calculated as the number of **T** and **T KO** divided by the number of treated cells (listed in column five) using the linear DNA cassette for *DUR3* targeting. The amount of the plasmid (400 ng) and of linear DNA (5 µg) was the same in all the experiments. For each strain one experiment of transformation with the plasmid and one with linear DNA was performed. The real disruptants were verified by colony PCR as schematically illustrated in Fig. S1. The νt and νko values were used to obtain the histograms represented in Figure S1. The mutant *rad52Δ/rad52Δ* was used as a negative control; since it did not show any homologous integration, it was not inserted in Fig. S1. Statistical meaning of the data is included in the Materials & Methods section.
(DOC)

Table S2 a) Number of transformations performed, average number of treated cells per transformation, transformants and integrants obtained in the wild type San1 and in the various deletants with a BIT cassette. The term “ectopics” refer to randomly integrated transformants, regardless the homology; adh1-int or dur3-int refer to transformants obtained with a cassette correctly integrated at one side only (*adh1* or *dur3,* respectively) and ectopically integrated on the other side. Translocants are transformants obtained by correct integration of the cassette at both *adh1* and *dur3 loci*. The distribution of events is also illustrated for *RAD54*, *RDH54* and *POL32* mutants in Fig. 1 (pies on the top, right). Raw data are reported also for *rad52Δ/rad52Δ,* used as negative control. Since homologous integrations in GT and in BIT for this mutant, as expected, were never obtained, the results were not plotted in Fig.1. **b)** **Computed values (νx/νp) used to generate** **Figure 1** **.** Frequencies (**νx**) related to strain transformability (**νp**). In particular, x indicates the frequency of transformation (**νt**), frequency of integration in *adh1* (**νiadh**), in *dur3* **(νidur**), in ectopic sites (**νect**), of translocation (**νTsl**) for the wild type San1 and for each mutant. The frequencies were obtained as number of transformants on G418 divided by the number of treated cells. The number of transformants per each event and strain is summarized in Table S2. The average number of treated cells per each transformation with a BIT cassette varied between 2.2 and 3.4×10^8^ and the exact amount is reported in Table S2. The **νp** values are summarized in Table S1. The number of independent determinations to reach the same amount of transformants is given in Table S2 (number of transformations). The standard error is indicated (±) next to each value in Table S3 and it is presented in Fig. 1. * = one clone only was recovered in this experiment.
(DOC)

Table S3 **Primers used in this work.** For each primer, the chromosomal location and the coordinates are indicated in brackets; the primers specific for the three BIT cassettes (VIII–XV; VIII–VIII; IX–XVI) are already reported in references 8, 9 and 10, respectively. Primers used only for sequencing and verification of the constructs are not reported in this list.
(DOC)
