# Supplementary figures and images for: Bridge-Induced Chromosome Translocation in Yeast Relies upon a Rad54/Rdh54-Dependent, Pol32-Independent Pathway
Source: PLoS One. 2013 Apr 17;8(4):e60926. doi: 10.1371/journal.pone.0060926 (PMC3629078; doi:10.1371/journal.pone.0060926)

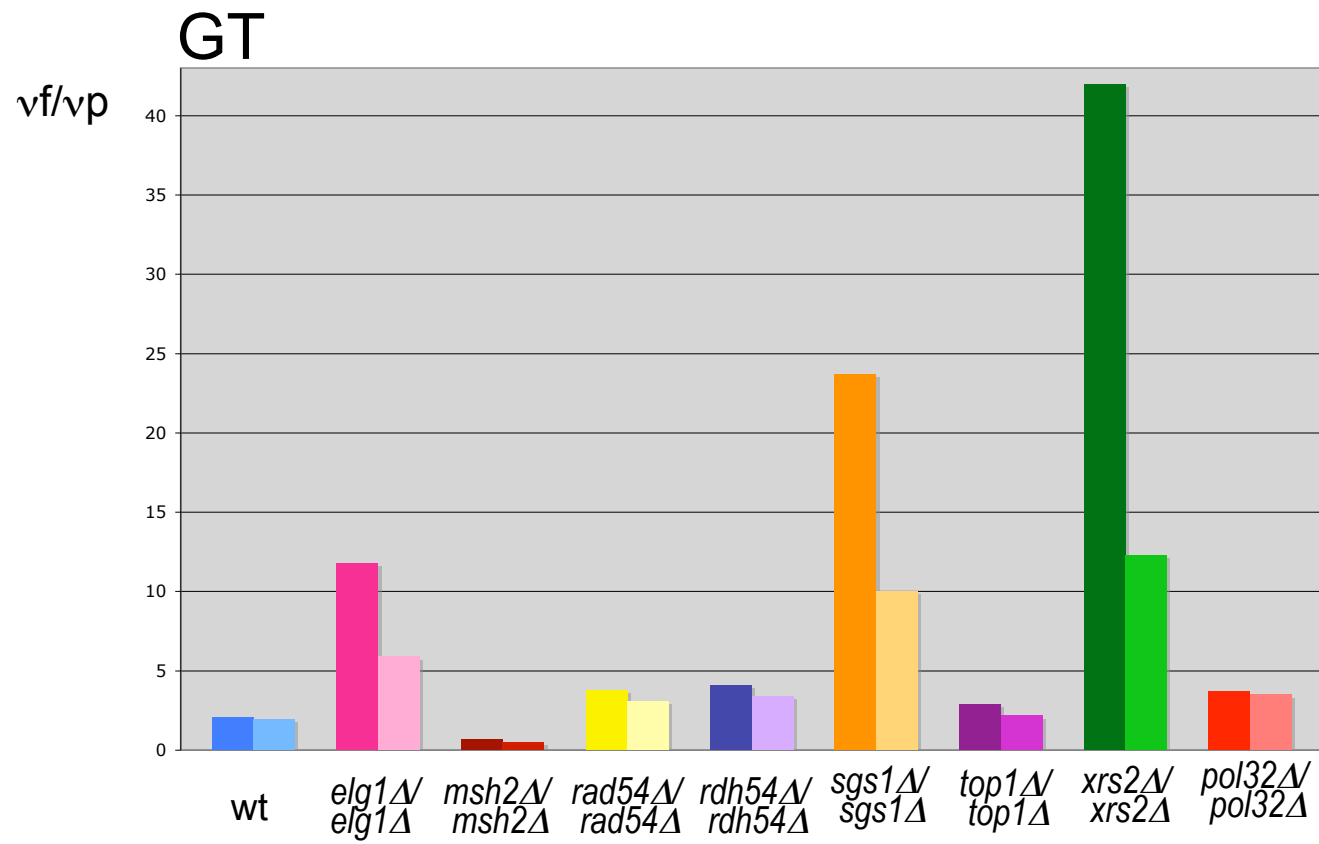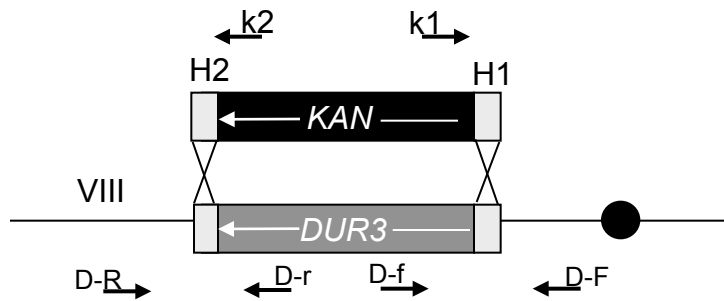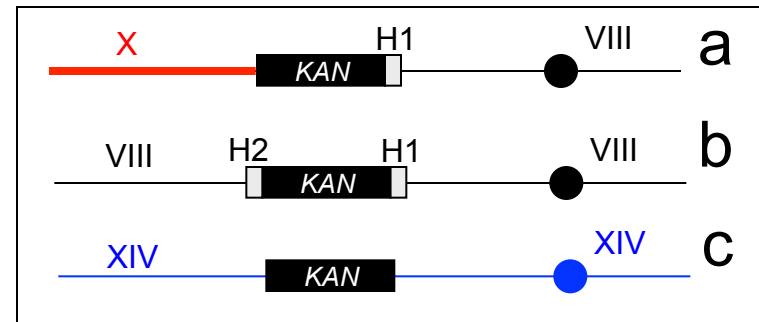

Supplement: Figure S1 — Histograms representing different transformation and knock out frequencies with a GT cassette. Frequency of transformation νt (left, dark bar) and of knock out νko (right, light bar) of the gene DUR3 in the wt and in eight different mutants divided by the transformability of each strain ( ν p). νf (f = fragment) in the y-axis indicates νt or νko for the left and the right bar respectively. Computational data used to draw the histogram are reported in Table S1. At the bottom, a scheme of the knock out with the relative homologies (H = 65 nt) is shown. The arrows indicate the orientation of the gene transcription. The homology H1 was also used with the BIT cassette (Figure 1). The primers (k1, D–F, D–f; k2, D–R, D–r) used for the verification of the integration by colony-PCR are also reported (for the primer sequences see Table S3). In the frame an example of one-end integration (a), two-end integration = KO (b) and ectopic integration (c) is illustrated. Ectopic integrations, regardless the homology, might occur also following integrations of the two ends into two different chromosomes (they generate in this case ectopic translocants). H1 and H2 in ectopic integrations and in one-end integrations may be completely or partially lost (degraded) as we previously reported [8]. (PDF) [file pone.0060926.s001.pdf]

# A

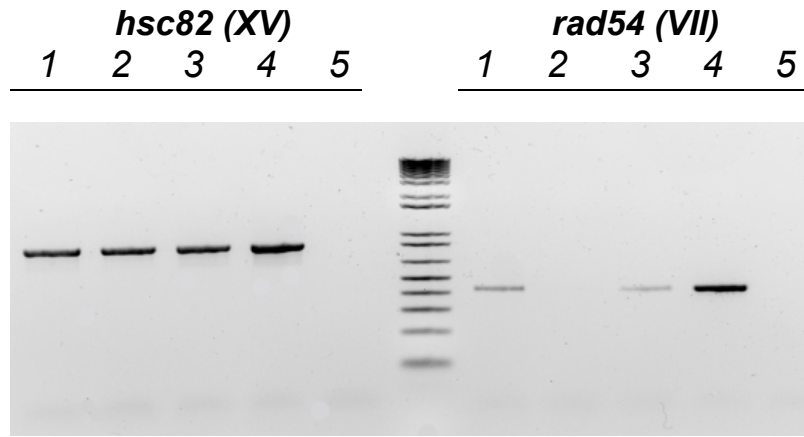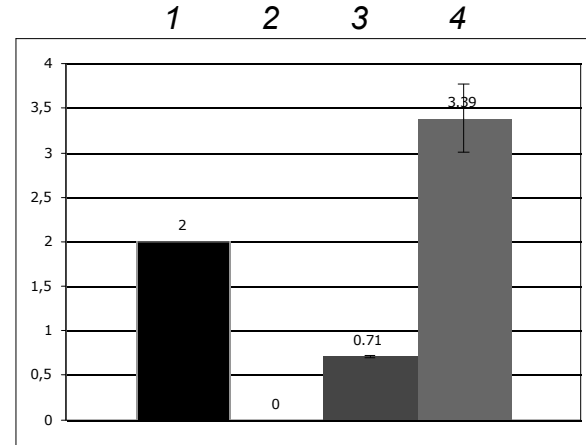

# B

| strain              | N trasformants | adh1-int | dur3-int | ectopics | N traslocants |
|---------------------|----------------|----------|----------|----------|---------------|
| <i>CRAD54</i>       | 57             | 27       | 1        | 27       | 2             |
| <i>OeRAD54</i>      | 107            | 65       | 1        | 38       | 3             |
| <i>RAD54/rad54Δ</i> | 51             | 22       | 4        | 19       | 6             |
| San1                | 51             | 21       | 9        | 18       | 3             |

Supplement: Figure S2 — RT-PCR analysis of RAD54 expression in the complementing (CRAD54) and over-expressing (OeRAD54) strain with the distribution of BIT integration events. The primers used to test RAD54 expression are listed in Table S3. A) Lane 1: wild type San1, 2: rad54Δ/rad54Δ, 3: CRAD54, 4: OeRAD54, 5: PCR negative control. HSC82 is a gene constitutively expressed, used as expression reference. On the right, the semi-quantitative RT-PCR analysis is quantified by laser-scanning densitometry as described in the Materials & Methods section. The results are plotted as histogram bars relative to two copies of RAD54 present in the diploid wild type on lane1. B) Number of transformants, translocants and one-end integrants (either in the adh or in the dur locus), in CRAD54, OeRAD54, the heterozygous strain (RAD54/rad54Δ) and in the wild type strain San1. (PDF) [file pone.0060926.s002.pdf]

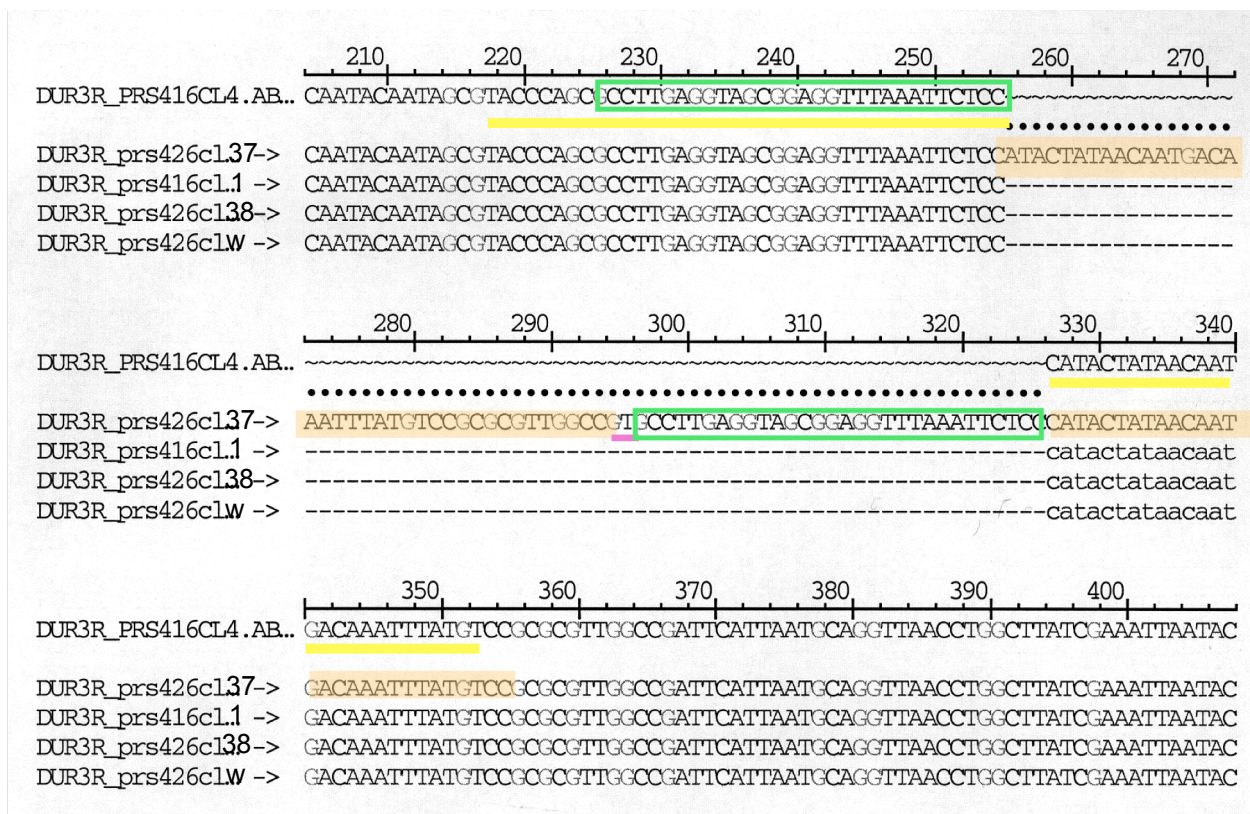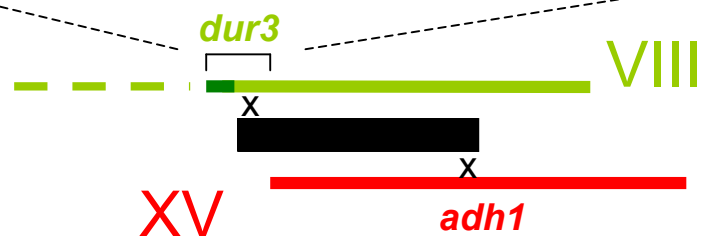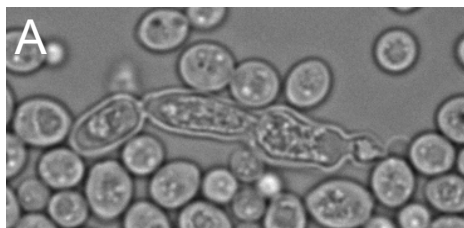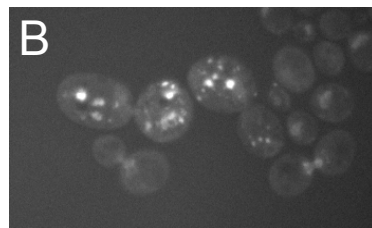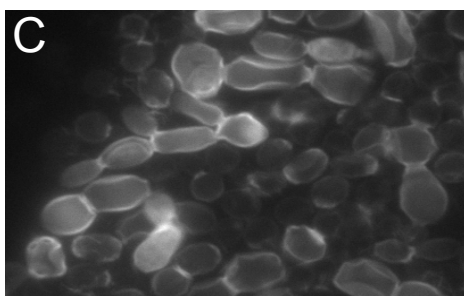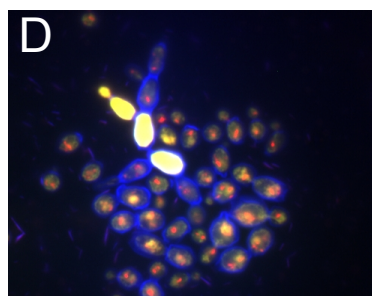

Supplement: Figure S3 — Genetic and phenotypic characterization of OeRAD54cl37. The first line on the top represents the consensus among the three OeRAD54 translocants and one translocant complementing RAD54 (CRAD54cl1) used as control. The 65 nt-homology used in the BIT event (H1 in the scheme of Figure 1) is underlined in yellow. The dots indicate the duplication (which is present only in OeRAD54cl37). A segment of 40 nt is outlined in orange and another one, consisting of 30 nt, is squared in green to highlight the origin of the duplication. One T (underlined in pink) was added by the cell within the two short duplicated segments. On the bottom of the panel, the phenotype of OeRAD54cl37 is shown: A) picture without staining evidencing the presence of dead ghost cells; B) DAPI staining showing nuclear fragmentation; C) calcofluor staining reveals unusual cellular shapes; D) FUN combined with calcofluor staining confirms the high cell mortality. (PDF) [file pone.0060926.s003.pdf]

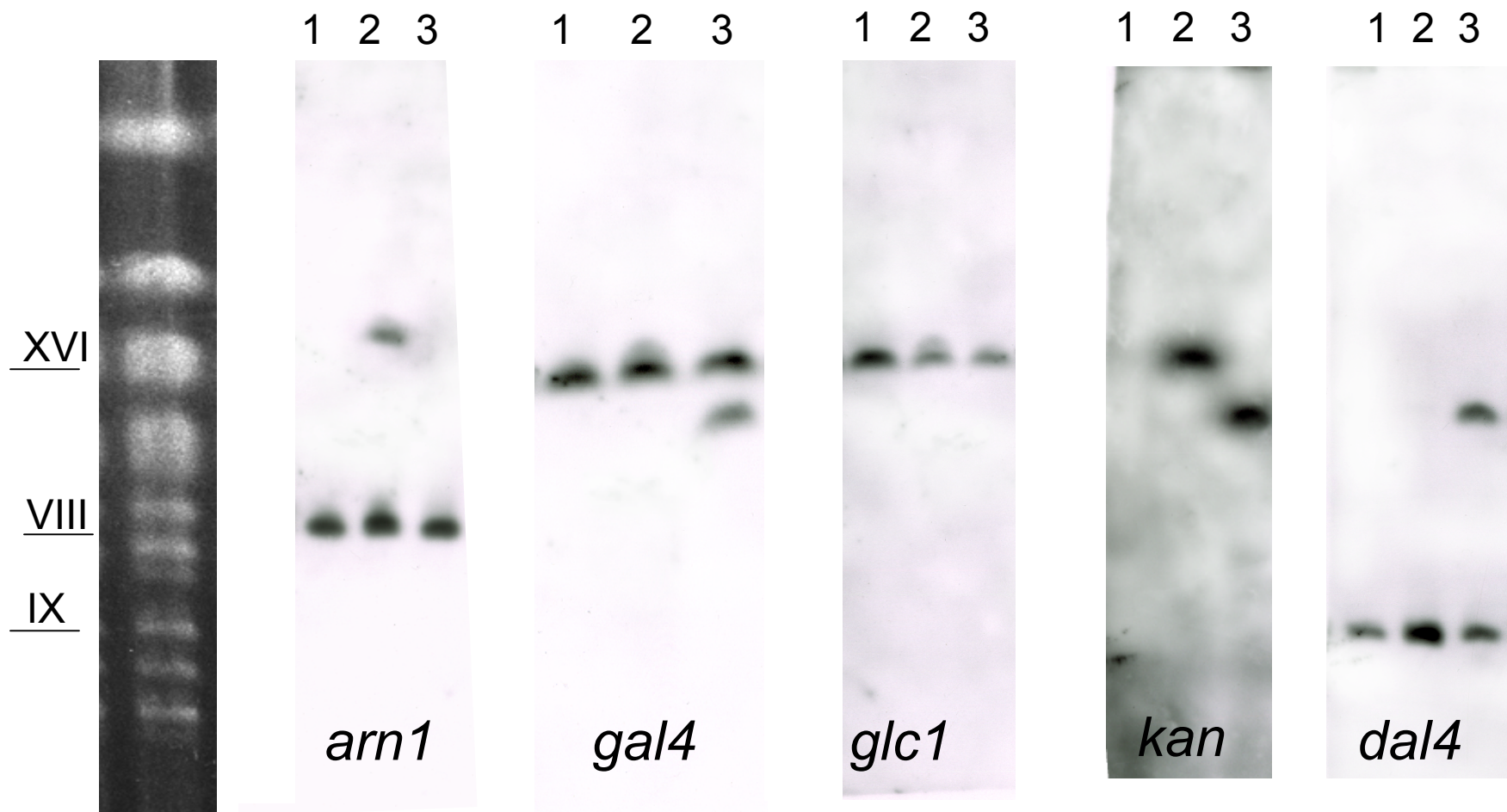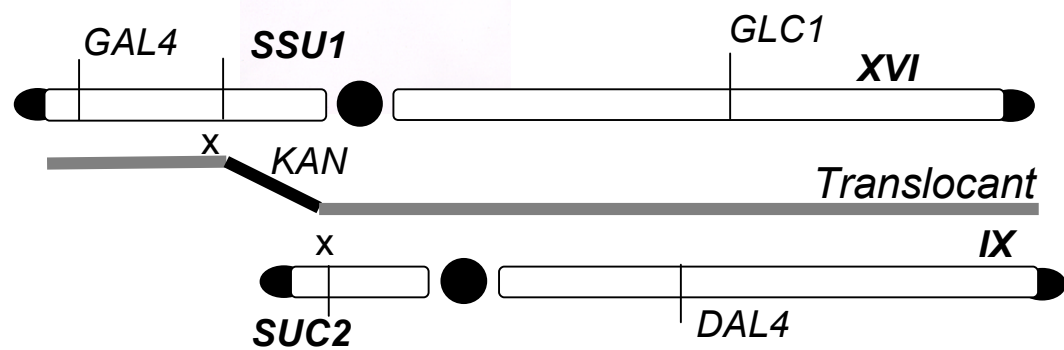

Supplement: Figure S5 — Southern hybridization analysis of the OeRAD54 translocant bridging chromosomes XVI and IX (SUSU). DNA hybridization with probes against the loci arn1, gal4, glc1, kan and dal4 (for primers see ref. 10) to verify the presence of both arms of the chromosomes. Lane 1: wild type San1; 2: AD translocant (XV–VIII) (used as hybridization control); 3: cl48 SUSUOeRAD54 (translocation IX–XVI). On the left, a lane of CHEF chromosomes separation indicates the chromosomes probed. The ARN1 gene is located on the left arm of chromosome VIII next to RIM4. The location of the other probes is indicated in the scheme below the hybridization panels. (PDF) [file pone.0060926.s005.pdf]

**A**

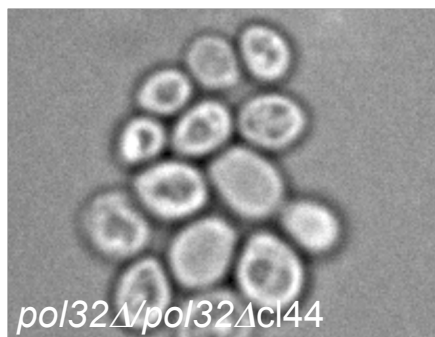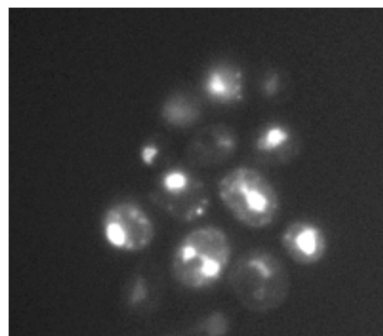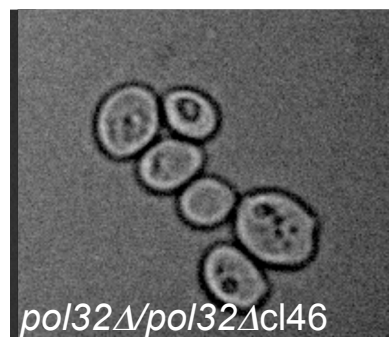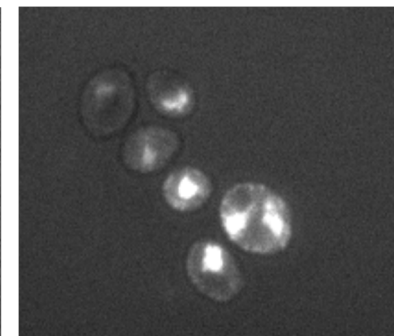

**B**

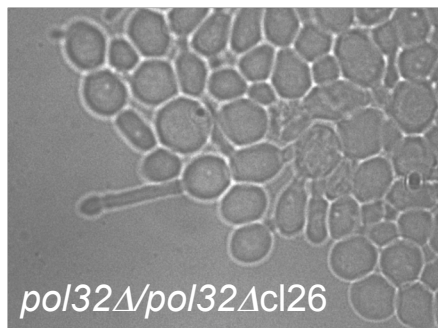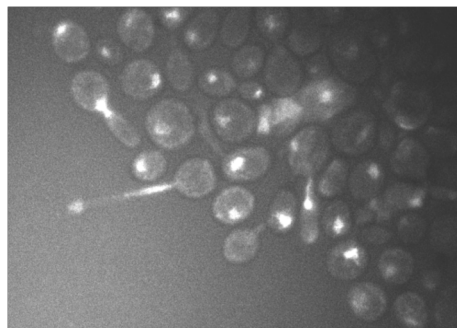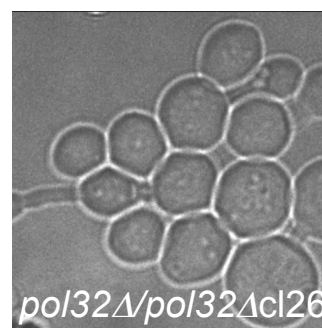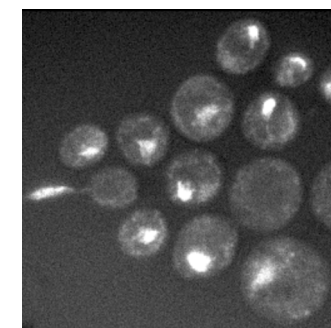

**C**

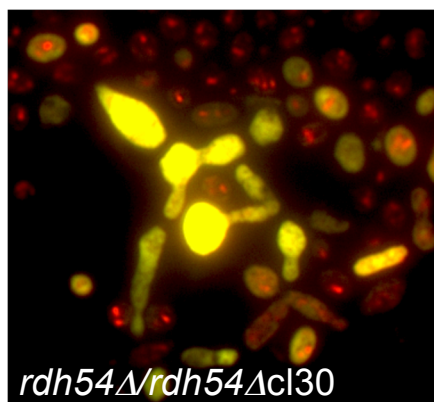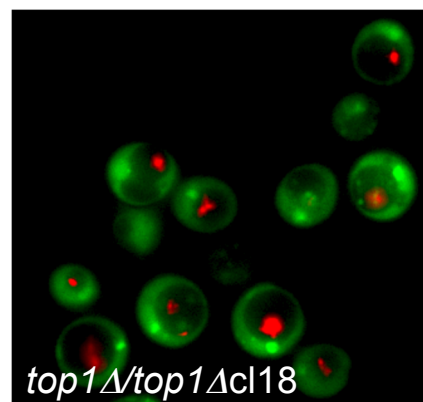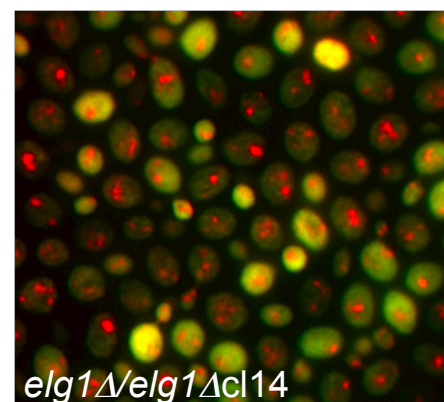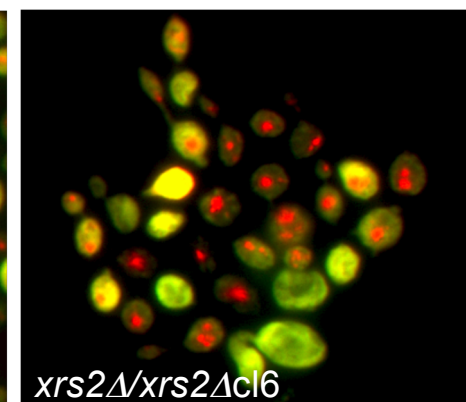

Supplement: Figure S6 — DAPI and FUN staining of several translocants showing peculiar phenotypes. A) Translocants number 44 and 46 in pol32Δ/pol32Δ without (left) and with (right) fluorescence microscopy after DAPI staining; single cells with two nuclei are visible. B) pol32Δ/pol32Δ cl26 is characterized by strong karyokynetic defects such as germination tube formation and by a strong flocculation. C) FUN staining of four translocants characterized by point mutations at the breakpoints; from left to right: rdh54Δ/rdh54Δcl30, top1Δ/top1Δcl18, elg1Δ/elg1Δcl14, xrs2Δ/xrs2Δcl6. Dead cells appear as yellow-colored. Point mutations, insertions or deletions around the breakpoints were never detected in the three translocants obtained in the wild type background. (PDF) [file pone.0060926.s006.pdf]
