# Supplementary material for: Bridge-Induced Chromosome Translocation in Yeast Relies upon a Rad54/Rdh54-Dependent, Pol32-Independent Pathway
Source: PLoS One. 2013 Apr 17;8(4):e60926. doi: 10.1371/journal.pone.0060926 (PMC3629078; doi:10.1371/journal.pone.0060926)
Supplement: Table S1 — Frequency of strain transformability with the tester plasmid Ycp50 (νp), frequency of strain transformation (νt) and of strain knock out (νko) with the linear cassette. The first column contains the number of transformants T obtained on -URA with YCp50 in each strain using always 1×10^7^ cells. The second column contains the frequency of strain transformability (νp) calculated as the number of transformants on –URA divided by the number of treated cells. The third column summarizes the number of transformants (T) obtained on G418 with the linear cassette. The fourth the number of real disruptants (T KO) with the linear cassette. The frequencies of transformation (νt) and of knock out (νko) are calculated as the number of T and T KO divided by the number of treated cells (listed in column five) using the linear DNA cassette for DUR3 targeting. The amount of the plasmid (400 ng) and of linear DNA (5 µg) was the same in all the experiments. For each strain one experiment of transformation with the plasmid and one with linear DNA was performed. The real disruptants were verified by colony PCR as schematically illustrated in Fig. S1. The νt and νko values were used to obtain the histograms represented in Figure S1. The mutant rad52Δ/rad52Δ was used as a negative control; since it did not show any homologous integration, it was not inserted in Fig. S1. Statistical meaning of the data is included in the Materials & Methods section. (DOC) [file pone.0060926.s007.doc]

**Table S1**

| **Strain** | **T**  **with YCp50** | **νp (x10^-5^)** | **T**  **with linear DNA** | **T KO** | **cells (x10^8^)**  **treated with linear DNA** | **νt/νp**  **(x10^-3^)** | **νko/νp**  **(x10^-3^)** |
| --- | --- | --- | --- | --- | --- | --- | --- |
| San1 | 937 | 9.4 | 35 | 34 | 1.78 | 2.1 | 2.0 |
| *elg1*Δ/*elg1*Δ | 115 | 1.2 | 24 | 12 | 1.70 | 11.8 | 5.9 |
| *msh2*Δ/*msh2*Δ | 1138 | 11 | 15 | 10 | 1.85 | 0.7 | 0.5 |
| *rad54*Δ/*rad54*Δ | 363 | 3.6 | 16 | 13 | 1.18 | 3.8 | 3.1 |
| *rdh54*Δ/*rdh54*Δ | 347 | 3.5 | 23 | 19 | 1.60 | 4.1 | 3.4 |
| *sgs1*Δ/*sgs1*Δ | 32 | 0.3 | 12 | 5 | 1.69 | 23.7 | 10 |
| *top1*Δ/*top1*Δ | 865 | 8.7 | 42 | 32 | 1.69 | 2.9 | 2.2 |
| *xrs2*Δ/*xrs2*Δ | 33 | 0.3 | 17 | 5 | 1.35 | 42 | 12.3 |
| *pol32*Δ/*pol32*Δ | 490 | 4.9 | 27 | 26 | 1.50 | 3.7 | 3.5 |
| *rad52*Δ/*rad52*Δ | 159 | 1.6 | 1 | 0 | 1.50 | 0.4 | 0 |
