# Supplementary material for: Bridge-Induced Chromosome Translocation in Yeast Relies upon a Rad54/Rdh54-Dependent, Pol32-Independent Pathway
Source: PLoS One. 2013 Apr 17;8(4):e60926. doi: 10.1371/journal.pone.0060926 (PMC3629078; doi:10.1371/journal.pone.0060926)
Supplement: Table S2 — a) Number of transformations performed, average number of treated cells per transformation, transformants and integrants obtained in the wild type San1 and in the various deletants with a BIT cassette. The term “ectopics” refer to randomly integrated transformants, regardless the homology; adh1-int or dur3-int refer to transformants obtained with a cassette correctly integrated at one side only (adh1 or dur3, respectively) and ectopically integrated on the other side. Translocants are transformants obtained by correct integration of the cassette at both adh1 and dur3 loci. The distribution of events is also illustrated for RAD54, RDH54 and POL32 mutants in Fig. 1 (pies on the top, right). Raw data are reported also for rad52Δ/rad52Δ, used as negative control. Since homologous integrations in GT and in BIT for this mutant, as expected, were never obtained, the results were not plotted in Fig.1. b) Computed values (νx/νp) used to generate Figure 1 . Frequencies (νx) related to strain transformability (νp). In particular, x indicates the frequency of transformation (νt), frequency of integration in adh1 (νiadh), in dur3 (νidur), in ectopic sites (νect), of translocation (νTsl) for the wild type San1 and for each mutant. The frequencies were obtained as number of transformants on G418 divided by the number of treated cells. The number of transformants per each event and strain is summarized in Table S2. The average number of treated cells per each transformation with a BIT cassette varied between 2.2 and 3.4×10^8^ and the exact amount is reported in Table S2. The νp values are summarized in Table S1. The number of independent determinations to reach the same amount of transformants is given in Table S2 (number of transformations). The standard error is indicated (±) next to each value in Table S3 and it is presented in Fig. 1. * = one clone only was recovered in this experiment. (DOC) [file pone.0060926.s008.doc]

**Table S2**

**a)**

| **Strain** | ***N transformations*** | ***N cells***  ***(x10^8^)*** | ***N transformants*** | **Integrants** | | | |
| --- | --- | --- | --- | --- | --- | --- | --- |
|  |  |  |  | **Ectopics** | **adh1-integrants** | **dur3-integrants** | **Tsl** |
| San1 | 3 | 3.46 | 51 | 18 | 21 | 9 | 3 |
| *elg1*Δ/ *elg1*Δ | 6 | 2.88 | 50 | 26 | 19 | 2 | 3 |
| *msh2*Δ/ *msh2*Δ | 8 | 2.88 | 52 | 19 | 26 | 7 | 0 |
| *rad54*Δ/ *rad54*Δ | 7 | 2.83 | 1 | 0 | 1 | 0 | 0 |
| *rdh54*Δ/ *rdh54*Δ | 5 | 3.30 | 51 | 19 | 15 | 11 | 6 |
| *sgs1*Δ/ *sgs1*Δ | 12 | 2.38 | 53 | 40 | 13 | 0 | 0 |
| *top1*Δ/ *top1*Δ | 7 | 2.48 | 50 | 27 | 16 | 3 | 4 |
| *xrs2*Δ/ *xrs2*Δ | 11 | 2.20 | 50 | 16 | 33 | 0 | 1 |
| *pol32*Δ/ *pol32*Δ | 28 | 2.48 | 48 | 31 | 6 | 2 | 9 |
| *rad52*Δ/ *rad52*Δ | 3 | 2.25 | 2 | 2 | 0 | 0 | 0 |

**b)**

|  |  |  | **x10^-3^** |  |  |
| --- | --- | --- | --- | --- | --- |
| **strain** | **νt/νp** | **νiadh/νp** | **νidur/νp** | **νect/νp** | **νTsl/νp** |
| San1(wt) | 0.52±0.19 | 0.22±0.09 | 0.09±0.04 | 0.18±0.09 | 0.03±0.03 |
| *elg1*Δ/ *elg1*Δ | 2.41±1.12 | 0.92±0.54 | 0.1±0.09 | 1.25±0.48 | 0.14±0.09 |
| *msh2*Δ/ *msh2*Δ | 0.21±0.05 | 0.10±0.02 | 0.03±0.01 | 0.075±0.04 | 0 |
| *rad54*Δ/ *rad54*Δ | 0.01* | 0.01* | 0 | 0 | 0 |
| *rdh54*Δ/ *rdh54*Δ | 0.88±0.14 | 0.26±0.08 | 0.19±0.07 | 0.33±0.04 | 0.10±0.03 |
| *sgs1*Δ/ *sgs1*Δ | 6.2±1.8 | 1.53±0.36 | 0 | 4.67±1.70 | 0 |
| *top1*Δ/ *top1*Δ | 0.33±0.08 | 0.11±0.02 | 0.02±0.01 | 0.18±0.06 | 0.03±0.017 |
| *xrs2*Δ/ *xrs2*Δ | 6.90±1.42 | 4.60±1.29 | 0 | 2.20±0.69 | 0.14* |
| *pol32*Δ/ *pol32*Δ | 0.14±0.03 | 0.02±0.01 | 0.006±0.004 | 0.09±0.02 | 0.03±0.01 |
