# Supplementary material for: Bridge-Induced Chromosome Translocation in Yeast Relies upon a Rad54/Rdh54-Dependent, Pol32-Independent Pathway
Source: PLoS One. 2013 Apr 17;8(4):e60926. doi: 10.1371/journal.pone.0060926 (PMC3629078; doi:10.1371/journal.pone.0060926)
Supplement: Table S3 — Primers used in this work. For each primer, the chromosomal location and the coordinates are indicated in brackets; the primers specific for the three BIT cassettes (VIII–XV; VIII–VIII; IX–XVI) are already reported in references 8, 9 and 10, respectively. Primers used only for sequencing and verification of the constructs are not reported in this list. (DOC) [file pone.0060926.s009.doc]

**Table S3**

*Primers used for the onstruction of the mutants (lower-case letters indicate the homology with kanamycin)*

**Fw1Elg1**(XV 605001-041):

GTGAGAAGACAAGATGCTCTACAGATTACCATCGATGACGAtaggcgtatcacgaggccc

**Rev1Elg1**(XV 602762-801):

CACGACTATGCTCGGATCAGCGTTGAACCATACGTTTTTCCatcgatgataagctgtcaaac

**Fw2Elg2**(XV 605052-092):

ATGAAAGGCACGTGTCTTTATCTGATATATTGACAGAAAtaggcgtatcacgaggccc

**Rev2Elg2**(XV 602717-756):

TTATTTGTTCTTTGAAAAGCCTGAGTGCAAATGCTCCCATCatcgatgataagctgtcaaac

**Fw1Msh2**(XV 147431-471):

GAAACTTCTATAAGAAGTATACAGGGTTGCCGAAGAAACCAtaggcgtatcacgaggccc

**Rev1Msh2**(XV:150179-218):

ATTTGCTATAGCACGCAATAGCTCTTGTATTTTATGCTGGCatcgatgataagctgtcaaac

**Fw2Msh2**(XV 147415-454):

ATGTATCAGAGGAGAGAAACTTCTATAAGAAGTATACAGGAtaggcgtatcacgaggccc

**Rev2Msh2**(XV 150237-276):

TTATAACAACAAGGCTTTTATATATTTCAGGTAATTATCGCatcgatgataagctgtcaaac

**Fw1Rad54**(VII 196320-360):

GTGAACGGCCGAGACTGGTACCTAGGCCTATTAACGTACAAtaggcgtatcacgaggccc

**Rev1Rad54**(VII 193797-836):

TGCGTCATGGTTCAAATGATTCCAAGTTGTCGCATCACCACatcgatgataagctgtcaaac

**Fw2Rad54**(VII 196370-409):

ATGGCAAGACGCAGATTACCAGACAGACCACCAAATGGAAAtaggcgtatcacgaggccc

**Rev2Rad54**(VII 193620-659):

CCATGCCCATCATTTAAAATAAATATGTAATGACCCCCCGCGatcgatgataagctgtcaaac

**Fw1Sgs1**(XIII 645147-186):

GACAAAGATTTCGTATTCCAGGCTATCCAAAAGCACATCGAtaggcgtatcacgaggccc

**Rev1Sgs1**(XIII 640999-38):

CCTTGTACCTGATTTGCTGCTTGTGGTATTTTTGGGTAATCatcgatgataagctgtcaaac

**Fw2Sgs1**(XIII 645228-270):

CACAAGGCGGTAATGGTGACGAAGCCGTCACATAACTTAAGAAAtaggcgtatcacgaggccc

**Rev2Sgs1**(XIII 640859-900):

GAATGCTTGGCGAATGGTGTCGTAGTTATAAGTAACACTATTAGCatcgatgataagctgtcaaac

**Fw1Top1**(XV 315307-047):

CTTCTGATGACGATGACGATGTGCCATTATCTCAAACTTTAtaggcgtatcacgaggccc

**Rev1Top1**(XV 313084-123):

CCTCCAATTTTCATCTACCGATTCTATGGCCCATTTGAATCatcgatgataagctgtcaaac

**Fw2Top1**(XV 315347-387):

ATGACTATTGCTGATGCTTCCAAAGTTAATCATGAGTTGTCAtaggcgtatcacgaggccc

**Rev2Top1**(XV 313011-050):

TAGTAACCCTAATGCGAACTTGATGCGTGAATGTATTTGCCatcgatgataagctgtcaaac

**Fw1Xrs2**(IV 1217493-533):

CTTTATATCATGTTGTCTTCAGGCCTTCAAAACTTATAGTAtaggcgtatcacgaggccc

**Rev1Xrs2**(IV 1215061-101):

GTCGTCGTCGCCATCACCGTTATTAGAAGCCCCAAAAGAGCatcgatgataagctgtcaaac

**Fw2Xrs2**(IV 1217541-580):

ATGTGGGTAGTACGATACCAGAATACATTGGAAGATGGCTAAAAtaggcgtatcacgaggccc

**Rev2Xrs2**(IV 1215016-055):

TTATCCTTTTCTTCTTTTGAACGTAAACTTCGGACCGTCGCatcgatgataagctgtcaaac

**Fw1Pol32**(X 517470-510):

GATCAAAAGGCGTCATATTTTATCAATGAGAAGCTCTTCACataggcgtatcacgaggccc

**Rev1Pol32**(X 516466-505):

TTGCCTTTCTTTTGAAAAAGCTTTCCAATGTTCCTTGCTTcatcgatgataagctgtcaaac

**Fw2Pol32**(X 517412-450):

TTTACGGATCTAATTCACCACTTGAAGATCGGCCCATCCataggcgtatcacgaggccc

**Rev2Pol32**(X 516524-563):

TGACGGCGTTTCTTGCTTTTTTGAAGACGACAATGCCCTTcatcgatgataagctgtcaaac

**Fw1Rdh54**(II 383169-208):

AACAGCTCGGTATATCAAACGGTATTTGATTCCGGTACTAtaggcgtatcacgaggccc

**Rev1Rdh54**(II 386080-19):

TGAATCAGCAATAGGCCCTACTTAATATGTGACTTGAACCatcgatgataagctgtcaaac

**Fw2Rdh54**(II 383258-297):

aagggttggatcaaataagtacacacaactcaaaccaacctaggcgtatcacgaggccc

**Rev2Rdh54**(II 385901-940):

AATACTATCTTTCAATTCGGTGAACGAATCTGTGATAACCatcgatgataagctgtcaaac

**Fw1Rad52** (XIII 213890-930):

ATGAATGAAATTATGGATATGGATGAGAAGAAGCCCGTTTTataggcgtatcacgaggccc

**Rev1Rad52**(XIII 212515-554):

TCAAGTAGGCTTGCGTGCATGCAGGGGATTGATCTTTGGTcatcgatgataagctgtcaaac

**Fw2Rad52** (XIII 213809-849):

AAATTAGGACCTGAGTATATCTCCAAGAGAGTTGGGTTTGGataggcgtatcacgaggccc

**Rev2Rad52**(XIII 212580-619):

TTTGTTGCGGAACGGCTGGCACTGCACCATTTCCATTAGGcatcgatgataagctgtcaaac

*Primers used for the amplification of the cassette with 400nt of homology:*

**UpR-PvuII:** TGTTGCATGAAGATACGTCTTG (VIII: 74373-394)

**UpF-BAMHI:** CGCGGATCCGCGGACGTTAACAATGTAGAACACC (VIII: 74012-033)

**DownF-EcoRV:** GCAGTAGTGACCCAAAAAATAG (VIII: 34357-378)

**DownR-EcoRI:** CGGAATTCCGGGCGTGTTTAACGATGTTTGA (VIII: 34768-788)

**FW400:** CATGAAGATACGTCTTGTTCG (VIII: 74369-389)

**REV400:** AGTAGTGACCCAAAAAATAGTG (VIII: 34359-380)

*Primers for RAD54 cloning and expression:*

**FW-RAD54:** CTTCATGTGCTTGCATGTGATG (VII: 196963-984)

**REV-RAD54:** AGAGATATTTACCAATTTGGCCC (VII: 193554-576)

**FW-RT-RAD54:** CTTAAGTTCTAAAGCAGGTGGG (VII: 193798-819)

**REV-RT-RAD54:** GCGTCATGGTTCAAATGATTCC (VII: 194202-223)

*Couples of primers used to generate probes and for DNA copy-number analysis (the sequence of the other probes cited in this work are reported in Table1 of Tosato et al., 2009):*

**FWACT1:** TGAAGCTCAATCCAAGAGAGGTATC (VI: 54196-220)

**REVACT1:** TTTGTTGGAAGGTAGTCAAAGAAGC (VI: 53325-349)

**FW BRX1:** CGGATTATTGCCTCATTCCAG (XV: 186545-565)

**REV BRX1:** TCTTACGACGTTTGGCGAAAC (XV: 185977-997**)**

**FW HAL9:** CACCCTTATGACAGGTAGTAC (XV: 153264-284)

**REV HAL9:** CAAGTCTAGCCACACTGTCAA (XV: 152701-721)

**FW ITR2:** ACTGATCAATCTTCTACGCAG (XV: 124100-120)

**REV ITR2:** TTAGCGCACTCGATATGTAAC (XV: 124383-403)

**FW SHR5:** AGATAACGCAAATACGAGCGA (XV: 109202-222)

**REV SHR5:** CCGTAAGCATATCGAGCAAC (XV: 109679-698)

**FW ANS1:** CACATTGTTTGCCATCACCAA (VIII: 360141-161)

**REV ANS1:** ATTCAACCTTCATACCAGCAC (VIII: 359752-772)

**FW NDJ1:** CCACCTTGGTTAATGTCGTC (XV: 117071-090)

**REV NDJ1:** AGTTCCCTGAGAACATTCGG (XV: 116760-779)

**FW ARN1:** GAATGGGTGATGTTGCTCTA (VIII: 19385-404)

**REV ARN1:** CAACTGGCCAATCGATGTTA (VIII: 18803-822)

**FW DCD1:** GAAGGAGACAGAGCTTGATT (VIII: 388313-332)

**REV DCD1:** TTCCGTAAAGCTGAATTGCC (VIII: 387812-831)

**FW CDC23:** AACAAAGATGGGGATGGTAG (VIII: 438575-594)

**REV CDC23:** TCGTTCGTTGACTACCAAGA (VIII: 438183-202)

**FW YDR262w:** ATCTACCTGTTAGTGTGCTGC (IV: 993144-164)

**REV YDR262w:** CGTTGGGGTCACTTACTTTG (IV: 993517-536)

**FW TyUP:** CTGGCATGAAGATTGGTATG (IV: 980305-324)

**REV TyUP:** TATCGCATTTCGGAAGTTGC (IV: 980660-679)

**FW TelR:** GTTGCATTGCCCTTACAGTT (VIII: 550663-682)

**REV TelR:** CTGAGTTACCTCAAACGGAC (VIII: 550183-202)

**FW TelL:** TACTTGGGGTAAGAATACTG (VIII: 7789-808)

**REV TelL:** GTGTAGTGATATGATTTGCGA (VIII: 7963-983)

**FW YDR213w:** TGATCTGTTCTGAGCCAAAG (VIII: 541074-093)

**REV YDR213w:** GATCCAGTGACAATGAAGCA (VIII: 541521-540)

**FW ADY4:** ACGTGAGCCGACAATTTGTG (XII: 592047-066)

**REV ADY4:** GCACAGTTGTTTCACTCCAG (XII: 592438-457)

**FW ECM22:** AATTCCCCTAATAGCGTTCC (XII: 602006-025)

**REV ECM22:** CCGAGGCCAAATAACTCTTG (XII: 601505-524)

**FW YLR257w:** TAATGAGGAAAGTGAGGCTTC (XII: 658924-944)

**REV YLR257w:** AATGTGGCCTGTTGGAATTG (XII: 659380-399)

**FW HAP1:** ACTGGTATGCGTGAATCATC (XII: 649589-608)

**REV HAP1:** TTGTCACCAGCAAGAAATCC (XII: 649955-974)

**FW CRP1:** ACTGAAGGTGTATTAGACGG (VIII: 391329-348)

**REV CRP1:** GGAGAAGCATTGCAACCAG (VIII: 392013-031)

**FW ELG1:** GCTTAATGCAAGGTTGCAGA (XV: 602595-614)

**REV ELG1:** AAGTGACGCCAATGTCAAGA (XV: 602896-915)

**FW RSC2:** TAGGTGCTCTTGCACTTGGT (XII: 841128-147)

**REV RSC2:** ATTTCTCAAGGACTAAGGCG (XII: 841645-664)

**FW SWR1:** AAAACCAACCTTGATCGCGC (IV: 1140718-737)

**REV SWR1:** ATAGCAGATGCAGATGTGGC (IV: 1140216-235)

**FW KIC1:** TTCCATTATGATCCAAAGGC (VIII: 316852-871)

**REV KIC1:** AGTCAAAGAAGAACAGAGGC (VIII: 316374-393)

**FW ARN2:** ACCGGGTGTTTGTATATAGC (VIII: 10697-716)

**REV ARN2:** AACTGGGTAACGCTACTGAA (VIII: 11113-132)

**FW RAD9:** TTGGGACTTCCTTCGATTATG (IV: 903308-328)

**FW RAD9:** TGCAGTTCTGATTAAACACGC (IV: 903588-608)
