# Supplementary material for: Bridge-Induced Chromosome Translocation in Yeast Relies upon a Rad54/Rdh54-Dependent, Pol32-Independent Pathway
Source: PLoS One. 2013 Apr 17;8(4):e60926. doi: 10.1371/journal.pone.0060926 (PMC3629078; doi:10.1371/journal.pone.0060926)
Supplement: Figure S4 — Analysis of the junction between chromosomes VIII and IV in rdh54 Δ/ rdh54 Δ. A) Scheme of the regions involved in the rearrangement; the resulting chromosome is drawn as filled color. Two asterisks indicate the breakpoints; the primers, used to amplify the region (DCD1-rev and YDR262-rev), are also reported. The loci rim4 and cdc23 are indicated in the upper scheme (chromosome VIII); the locus act1 (chromosome VI) was used as internal control. The junction between chromosomes VIII and IV was successfully amplified using the primers REV DCD1 and REV YDR262w (Table S3) and sequenced (B). It revealed a complex rearrangement in rdh54Δ/rdh54Δ background involving two LTRs sharing strong similarity (C). On chromosome VIII the break is within the LTRΔ10 while on chromosome IV it takes place within LTRΔ21. B) The sequencing electropherogram of the junction IV/VIII is reported in details. The switching between the two chromosomes is highlighted. C) BLAST (Basic Local Alignment Search Tool) of the two LTRs where the translocation took place. The query corresponds to a sequence region of chromosome VIII within LTRΔ10 (SGD coordinates: 389177–389400) and the subject corresponds to a region of chromosome IV of the LTRΔ21 (SGD coordinates: 987150–987380). The alignment allows the visualization of the minor differences between the two LTRs and it allows the comparison (in yellow) with the real, hybrid sequence (B) at the point of the junction. (PDF) [file pone.0060926.s004.pdf]

A

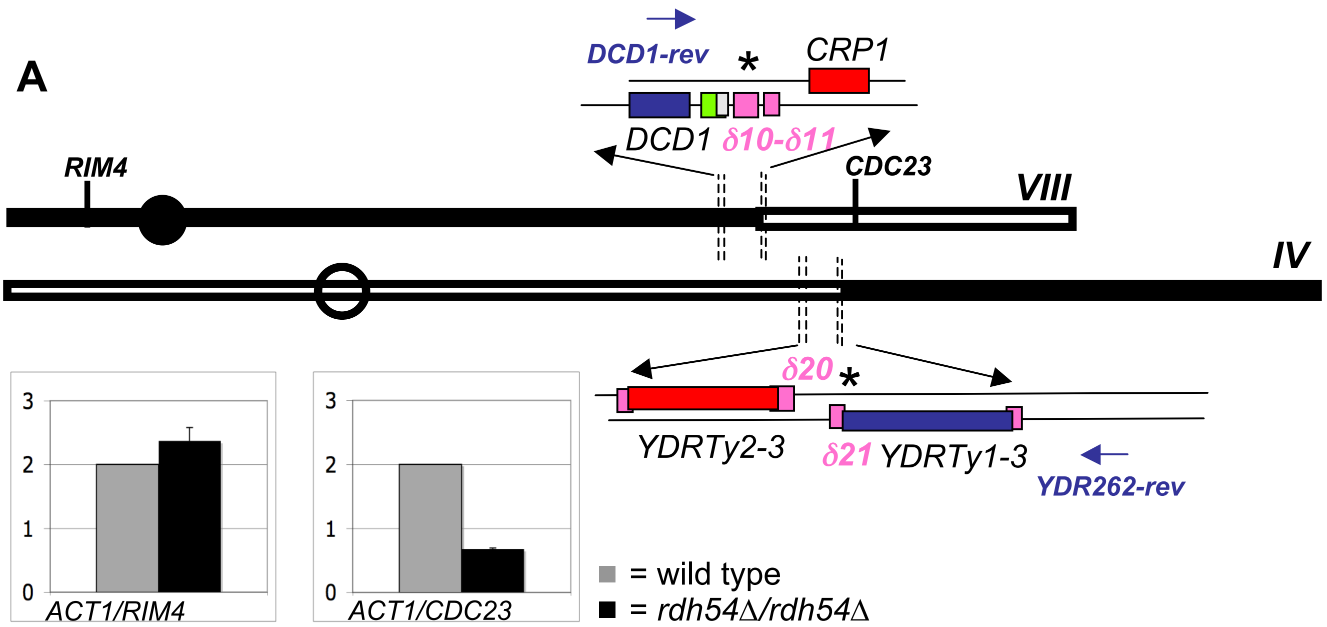

B

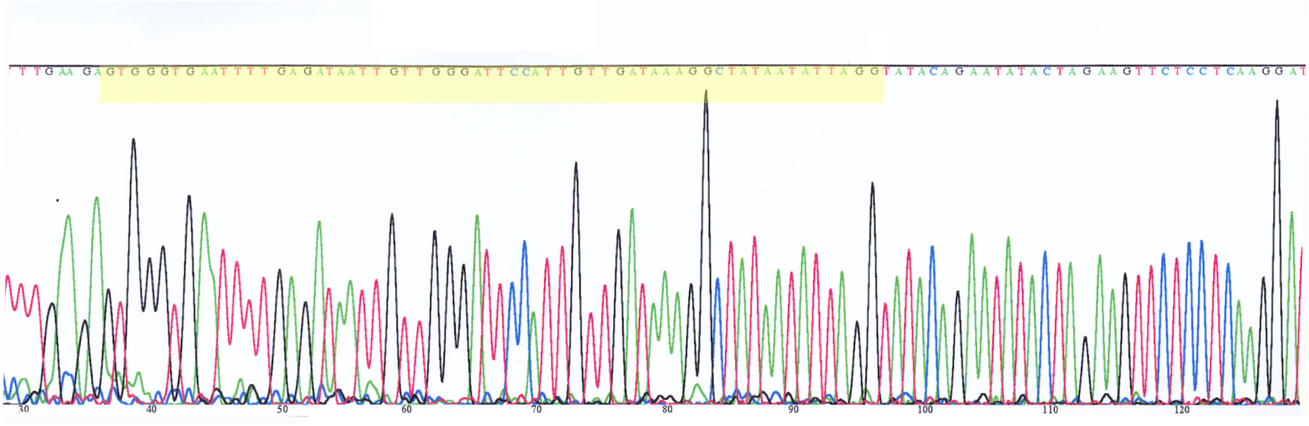

C

```
Score =  333 bits (368),  Expect = 3e-96
Identities = 207/219 (95%), Gaps = 5/219 (2%)
Strand=Plus/Plus

Query  7    GTGGGTGAATTTTGAGATAATTGTTGGGATTCCATTTTTAATAA-GGCAATAATATTAGG  65
            |||||| ||||    |||||||||||||||||||||| || |||| ||| |||||||||||
Sbjct  11   GTGGGT-AATT---AGATAATTGTTGGGATTCCATTGTTGATAAAGGCTATAATATTAGG  66

Query  66   TATGTAGAATGTACTAGAAGTTCTCCTCGAGGATTTAGGAATCCATAAAAGGGAATCTGC  125
            |||  ||||| ||||||||||||||||||||||||||||||||||||||||||||||||||
Sbjct  67   TATACAGAATATACTAGAAGTTCTCCTCGAGGATTTAGGAATCCATAAAAGGGAATCTGC  126

Query  126  AATTCTACACAATTCTATAAATATTATTATCATCATTTTATATGTTAATATTCATTGATC  185
            |||||||||||||||||||||||||||||||||| |||||||||||||||||||||||||
Sbjct  127  AATTCTACACAATTCTATAAATATTATTATCATCGTTTTATATGTTAATATTCATTGATC  186

Query  186  CTATTACATTATCAATCCTTGCGTTTCAGCTTCCACTAA  224
            |||||||||||||||||||||||||||||||||||||||
Sbjct  187  CTATTACATTATCAATCCTTGCGTTTCAGCTTCCACTAA  225
```
